# Tolvaptan versus fluid restriction in acutely hospitalised patients with moderate-profound hyponatraemia (TVFR-HypoNa): design and implementation of an open-label randomised trial

**DOI:** 10.1186/s13063-022-06237-5

**Published:** 2022-04-21

**Authors:** Annabelle M. Warren, Mathis Grossmann, Rudolf Hoermann, Jeffrey D. Zajac, Nicholas Russell

**Affiliations:** 1 Department of Medicine, University of Melbourne, Melbourne, Victoria Australia; 2 Department of Endocrinology, The Austin Hospital, Melbourne, Victoria Australia

**Keywords:** Hyponatraemia, Tolvaptan, Fluid restriction, Syndrome of inappropriate antidiuresis (SIAD)

## Abstract

**Background:**

Current hyponatraemia guidelines are divided on the use of tolvaptan in hospitalised patients with moderate to severe hyponatraemia, due to an uncertain risk-benefit ratio. We will conduct a randomised trial to test the hypothesis that early use of tolvaptan improves the rate of serum sodium correction and clinical outcomes compared with current standard first-line therapy, restriction of fluid intake, without increasing the risk of serum sodium overcorrection.

**Methods:**

We will enrol hospitalised patients with euvolaemic or hypervolaemic hyponatraemia and serum sodium of 115–130 mmol/L at Austin Health, a tertiary care centre in Melbourne, Australia. Participants will be randomised 1:1 to receive either tolvaptan (initial dose 7.5 mg) or fluid restriction (initial limit 1000 ml per 24 h), with titration of therapy based on serum sodium response according to a pre-determined protocol over a 72-h intervention period.

The primary endpoint will be the between-group change in serum sodium over time, from study day 1 to day 4. Secondary endpoints include serum sodium increment in the first 24 and 48 h, proportion of participants with normalised serum sodium, length of hospital stay, requirement for serum sodium re-lowering with intravenous dextrose or desmopressin, cognitive and functional measures (Confusion Assessment Method Short form, Timed Up and Go test, hyponatraemia symptom questionnaire), 30-day readmission rate, treatment satisfaction score and serum sodium 30 days after discharge. The trial will be overseen by an independent Data Safety Monitoring Board. Serum sodium will be monitored every 6–12 h throughout the study period, with pre-specified thresholds for commencing intravenous 5% dextrose if serum sodium rise targets are exceeded.

**Discussion:**

We seek to inform future international guidelines with high-quality data regarding the utility and safety of tolvaptan compared to standard therapy fluid restriction in patients with moderate-severe hyponatraemia in hospital. If tolvaptan use in this patient group is endorsed by our findings, we will have established an evidence-based framework for tolvaptan initiation and monitoring to guide its use.

**Trial registration:**

Australia and New Zealand Clinical Trials Registry ACTRN12619001683123. Registered on December 2 2019

**Supplementary Information:**

The online version contains supplementary material available at 10.1186/s13063-022-06237-5.

## Administrative information

Note: the numbers in curly brackets in this protocol refer to SPIRIT checklist item numbers. The order of the items has been modified to group similar items (see http://www.equator-network.org/reporting-guidelines/spirit-2013-statement-defining-standard-protocol-items-for-clinical-trials/).

**Table:** 

Title {1}	Tolvaptan versus fluid restriction in acutely hospitalised patients with moderate-severe hyponatraemia (TVFR-HypoNa): Design and implementation of an open-label randomised trial
Trial registration {2a and 2b}.	Australia and New Zealand Clinical Trials Registry ACTRN12619001683123. Registered 2 December 2019.
Protocol version {3}	16 April 2021, Version 16
Funding {4}	Investigator-initiated trial. Otsuka Australia Pharmaceutical (maker of tolvaptan) have agreed to provide financial support to reimburse study drug purchase and expenses. National Health and Medical Research Council of Australia has awarded a postgraduate research scholarship to AMW for financial support during PhD candidature.
Author details {5a}	Dr Annabelle M Warren MBBS Corresponding author – annabelle.warren@austin.org.au Prof Mathis Grossmann PhD Prof Rudolf Hoermann PhD Prof Jeffrey D Zajac PhD Dr Nicholas Russell MBBS *Affiliations above*
Name and contact information for the trial sponsor {5b}	The University of Melbourne Department of Medicine, Austin Health Level 7, Lance Townsend Building 145 Studley Rd, Heidelberg Victoria, 3084, Australia
Role of sponsor {5c}	The sponsor (The University of Melbourne) and the funding sources (Otsuka Australia Pharmaceutical and National Health and Medical Research Council of Australia) do not have any role in study design, data collection, analysis or interpretation, nor in decision to submit the report for publication.

## Introduction

### Background and rationale {6a}

Low serum sodium, known as hyponatraemia, is the most common electrolyte disorder, affecting 15–30% of acute hospital inpatients [1, 2]. Severe hyponatraemia can have serious acute complications due to cerebral oedema, and even mild to moderate hyponatraemia is associated with falls, delirium and increased length of hospital stay [3, 4]. Hyponatraemia at hospital admission is associated with increased inpatient mortality, though causation is not established [5, 6].

‘Severe’ hyponatraemia is defined by the presence of severe symptoms such as vomiting, seizure and reduced conscious state [7]. Biochemically, hyponatraemia can be classified as mild (Na 130–135 mmol/L), moderate (125–129 mmol/L) or profound (< 125 mmol/L) [7]. A critical consideration in the management of moderate-profound hyponatraemia of duration > 48 h (or unknown) is the need to avoid rapid correction which may lead to osmotic demyelination syndrome and permanent neurological disability. Guidelines recommended aiming to increase serum sodium by 5–8 mmol/L per day, and not to exceed 10 mmol/L per 24 h [7].

Current guideline-directed management of hyponatraemia is largely based on expert opinion and has limitations. Most cases of euvolaemic or hypervolaemic hyponatraemia are perpetuated by non-osmotic release of arginine vasopressin (AVP, also known as antidiuretic hormone (ADH)), causing water retention. This condition is known as syndrome of inappropriate antidiuresis (SIAD). Fluid restriction is the first-line treatment for patients with hyponatraemia who are not hypovolaemic [4, 7]. This treatment can be difficult to reliably administer because of the need for supervision, and for competing therapies requiring fluid administration. Fluid restriction is ineffective in approximately 50% of cases and may lead to reduced compliance and poor quality of life [8–10].

Tolvaptan is an oral vasopressin V2-receptor antagonist that blocks AVP action in the kidney, inducing a water diuresis to raise serum sodium concentration. The efficacy of tolvaptan versus placebo (without fluid restriction) was established in the SALT-1 and SALT-2 trials in patients with mild-to-moderate hyponatraemia (mean baseline serum sodium 129.0 (SD 4.1) mmol/L). The SALT trials did not enrol acutely hospitalised patients; rather, patients with chronic hyponatraemia were electively admitted for the purpose of trial initiation, and patients with serum sodium less than 120 mmol/L and neurological symptoms were excluded [11]. Tolvaptan was approved in the USA by the Food and Drug Administration in 2009 for treatment of clinically significant hypervolaemic or euvolaemic hyponatraemia, defined as serum sodium < 125 mmol/L, or hyponatraemia that is symptomatic and has resisted correction with fluid restriction [12, 13]. The sodium cut-off recommendation from the FDA approval is not clearly evidence-based and instead is based on expert opinion regarding which patients with hyponatraemia are likely to benefit from treatment in general [12].

Off-label experience with tolvaptan post-approval has identified benefits and risks. While the SALT trials reported excessively rapid correction of hyponatraemia (rise in sodium > 12 mmol in 24 h) in 1.7% of tolvaptan-treated patients (5.6% of an SIAD subgroup), observational studies of tolvaptan in moderate-to-profound hyponatraemia have shown highly variable rates of biochemical overcorrection in patients with initial serum Na < 125 mmol/L, ranging from 0 to 30% of cases [14–17]. An international hyponatraemia registry observed overcorrection of serum sodium in 12.1% of cases of tolvaptan use [9]. This experience has led some to recommend reduced initial dosing of tolvaptan (e.g. 7.5 mg) to improve its safety profile [18, 19]. At least one case of osmotic demyelination due to tolvaptan has been published, in a patient with decompensated cardiac failure in whom tolvaptan was inappropriately continued for several days despite a large initial rise in serum sodium [20, 21].

Currently, tolvaptan use in hospitalised patients with moderate to profound hyponatraemia is not evidence-based as there are no randomised controlled trials comparing the use of tolvaptan in hospitalised patients against standard of care fluid restriction. International guidelines are divided on the use of tolvaptan in hyponatraemia with lower sodium concentrations, due to concern regarding risk of sodium overcorrection [4, 7]. European guidelines recommend against the use of tolvaptan in moderate-profound hyponatraemia [7], whereas US/Irish guidelines acknowledge that there is insufficient evidence for tolvaptan use with serum sodium < 120 mmol/L, but endorse its use with appropriate monitoring of serum sodium levels, and prompt intervention if the recommended rate of serum sodium increase is exceeded [4]. Other potential concerns raised about tolvaptan include cost [22] and risk of liver function derangement seen with sustained higher-dose tolvaptan therapy for other indications (e.g. autosomal dominant polycystic kidney disease) [23].

Thus, there is a critical evidence gap with respect to efficacy and safety of tolvaptan in the very patients for whom it would be most useful. As such, there is physician reluctance to use what may be a safe and more efficacious therapy than fluid restriction if prescribed appropriately. There is an international need for rigorous randomised comparisons between tolvaptan and fluid restriction to determine evidence-based protocols for the use of tolvaptan in hospital inpatients with moderate to profound hyponatraemia, to clarify its role and ensure optimal patient outcomes.

### Objectives {7}

Aim: To compare the efficacy and safety of tolvaptan versus a rigorous, protocol-based fluid restriction to raise serum sodium levels in acute hospital inpatients with moderate-to-profound euvolaemic or hypervolaemic hyponatraemia.

Hypothesis: Tolvaptan will be more effective than fluid restriction in raising serum sodium and will lead to faster improvement in hyponatraemia-related symptoms, reduced length of hospital stay, without increasing adverse events.

### Trial design {8}

We will conduct a 3-day, parallel group, open-label, randomised, controlled trial of tolvaptan versus fluid restriction in hospitalised patients with moderate-to-profound hyponatraemia (Na 115–130 mmol/L). Patients will be randomised 1:1 to the tolvaptan or fluid restriction groups, with the intervention titrated daily based on serum sodium response according to a pre-determined protocol. The trial is designed to determine superiority. A 24–48h run-in period prior to randomisation will allow exclusion of patients with autocorrection of hyponatraemia due to an acutely reversible cause of non-osmotic AVP release.

## Methods: participants, interventions and outcomes

### Study setting {9}

We will enrol hospitalised patients with euvolaemic or hypervolaemic hyponatraemia, serum sodium 115–130 mmol/L at Austin Health, a tertiary care centre in Melbourne, Australia. Participants will be randomised 1:1 to receive either tolvaptan (initial dose 7.5 mg) or fluid restriction (initial limit 1000 ml per 24 h), with titration of therapy based on serum sodium response according to a pre-determined protocol over a 72-h intervention period.

### Eligibility criteria {10}

Inclusion criteria:

- Serum sodium 115–130 mmol/L on direct sodium measurement (venous blood gas), corrected for glucose level if glucose > 10 mmol/L [24]
- Age 18 years or over
- Informed consent (patient, or medical treatment decision maker)

Exclusion criteria:

- Hypovolaemia as defined as either:
  - Clinical impression of hypovolaemia; or
  - Urine sodium < 20 mmol/L (or urine chloride < 10 mmol/L in the presence of metabolic alkalosis) in the absence of pitting oedema.
- Polydipsia/polyuria, defined by first collected urine sample specific gravity < 1.003
- Severe symptoms of hyponatraemia warranting hypertonic saline:
- Vomiting (not attributable to other cause)
- Coma, defined by GCS < 8 on day 1 of admission
- Deep somnolence, defined by sedation score > 1 on day 1 of admission
- Seizure at any time in admission
- Respiratory arrest at any time in admission
- Thiazide or thiazide-like diuretic use within preceding 5 days
- Risk factors for osmotic demyelination syndrome:
  - Malnutrition (BMI < 16 or other clinical concern)
  - Alcohol abuse (> 14 standard drinks per week)
  - Child-Pugh B or C Cirrhosis
  - Hypokalaemia (≤ 3.0 mmol/L) on day 1, or need for intravenous potassium replacement
  - Increment in serum sodium from baseline to day 1 of > 0.5 mmol/L/h or > 10 mmol/L/24 h
- Other potential endocrine cause of hyponatraemia:
  - Untreated glucocorticoid deficiency or mineralocorticoid deficiency (evaluated with serum cortisol level, in context of clinical history and concurrent medications)
  - Significant hypothyroidism (TSH > 20 IU/L, and/or free T4 < 6 pmol/L)
- Chronic kidney disease stage 5 (estimated glomerular filtration rate (eGFR) < 15 ml/min/1.73 m^2^ or renal replacement therapy)
- Systolic blood pressure < 100 mmHg
- Inability to drink fluid unaided
- Pregnancy (by history, confirmed if necessary by serum *β*HCG) or breastfeeding
- Marked hyperglycaemia (day 1 venous blood gas glucose > 30 mmol/L)

### Who will take informed consent? {26a}

Participants will be approached on the hospital ward and given verbal explanation of the study by a study researcher, in most cases an endocrinologist or endocrinology specialist trainee doctor. A written participant information and consent form will be provided. Participants will be informed that their decision whether or not to participate in the study will not impact their access to routine care. Participants will be given the opportunity to read, discuss with support people and ask questions. Those willing to participate will sign the consent form.

For those patients who lack capacity to consent for themselves, the surrogate medical treatment decision maker will be identified and approached in person or by phone, and provided with the written participant information and consent form to assess and sign or not on behalf of the participant. Participants for whom consent is obtained from a medical treatment decision maker will be reassessed every 24 h. If the patient develops capacity to consent they will be provided with a continue to participate consent form and asked whether they consent to continuing to participate in the study.

### Additional consent provisions for collection and use of participant data and biological specimens {26b}—if applicable

Blood and urine biochemistry results will be derived from samples taken as part of routine hospital care. An additional sub-study is planned evaluating serum copeptin levels on day 1 and day 4, with serum samples be collected and stored for analysis with patient permission.

### Interventions

#### Explanation for the choice of comparators {6b}

We seek to determine the utility of tolvaptan in real-world practice through comparison with current standard therapy. While the original SALT trials of tolvaptan were placebo-controlled, these were conducted in ambulatory patients with chronic mild-moderate hyponatraemia (serum sodium 125–135 mmol/L) [11]. The choice of placebo or no intervention would not be a clinically useful or ethical comparison in our population of interest with moderate-to-profound hyponatraemia, as it may risk harm in these patients by delaying treatment.

To reflect real-world practice and for safety, both tolvaptan and fluid restriction will be titrated according to a protocol that allows for escalation and de-escalation of treatment according to serum sodium response in a pre-specified manner.

Tolvaptan (Samsca) is a selective vasopressin V2-receptor antagonist. It is approved in Australia for treatment of clinically significant hypervolaemic or euvolaemic hyponatraemia (serum sodium less than 125 mmol/L, or less marked hyponatraemia that is symptomatic and has resisted correction with fluid restriction) including patients with heart failure and syndrome of inappropriate antidiuresis (SIAD) [13]. In Australia, Tolvaptan is supplied by Otsuka Australia Pharmaceutical Pty Ltd. Detailed pharmacodynamic information, pharmacokinetic information, adverse effects, contraindications and warnings are available in Additional file 3: Appendix 3.

The SALT trials of Tolvaptan versus placebo utilised an escalating dose protocol, with initial dose 15 mg able to be increased to 30 mg then 60 mg in the event of inadequate initial response [11]. The proportion of patients requiring higher doses was not reported. We have chosen a lower starting dose of tolvaptan 7.5 mg in line with post-marking observational studies that have shown reduced rates of serum sodium rise > 12 mmol/24 h with this lower dose [17–19, 25]. While use of an even lower dose of tolvaptan 3.75 mg has been reported [15], the triangular shape in Australia of the lowest-available 15-mg tablet means that it cannot be accurately quartered, leading to selection of 7.5 mg as our starting dose to balance safety, efficacy and practical considerations.

Fluid restriction is the standard initial therapy for euvolaemic hyponatraemia, supported by international guidelines [4, 7]. While the quantity of fluid restriction is not always specified, an initial fluid restriction limit of 1.0 L (1000 ml) per 24 h is evidence-based [26]. Titration of the fluid restriction, in the form of pre-specified reduction in fluid restriction limit if sodium correction targets are not met, has been included in this protocol to reflect current clinical practice and ensure adequacy of treatment. US recommendations suggest down-titration of fluid restriction to 500 ml below 24-h urine output [4] and there is evidence for use of fluid restrictions in the range of 500–1000 ml [27]. We have opted for sequential restriction limits of 750 ml, 500 ml or 0 ml per 24 h total fluid intake for consistency.

#### Intervention description {11a}

Tolvaptan or fluid restriction will be administered and titrated according to serum sodium response (Table 1). In the tolvaptan group, the initial dose will be 7.5 mg, and in the fluid restriction group, the initial total fluid restriction will be 1000 mL.

**Table 1:** Dosing protocol for study interventions

DAY	Serum sodium	Prior 24 h sodium increment (mmol/L)	Tolvaptan (TOL) Group	Fluid Restriction (FR) Group	Sodium normalised protocol (both groups)
**1**	115–130		7.5 mg	1000 mL	
**2**	> 134	Any	➔	➔	1500 mL fluid restriction^a^
	≤134	> 8	Nil^b^	Nil^b^	
		5–8	7.5 mg	1000 mL	
		0–4	15 mg	750 mL	
		< 0	30 mg	500 mL	
**3**	> 134	Any	➔	➔	1500 mL fluid restriction^a^
	≤134	> 8	Nil^b^	Nil^b^	
		5–8	Same as D2	Same as D2	
		0–4	Dose increment^c^	FR increment^c^	
		< 0	Dose increment^c^	FR increment^c^	
**4**	Usual care after morning blood test including repeat serum sodium 30 days after discharge.				

^a^All participants regardless of group assignment with Na > 134 mmol/L on day 2 or on day 3 will receive a 1500-mL fluid restriction that day and no other intervention unless the Na increment was > 8 mmol/L over the preceding 24 h in which case they will not receive fluid restriction or tolvaptan

^b^Participants with Na increment > 8 mmol/L over the preceding 24 h will not receive a fluid restriction or tolvaptan for that day, regardless of group assignment

^c^Stepwise dose/fluid restriction increments:

• Tolvaptan: 7.5 mg ➔ 15 mg ➔ 30 mg ➔ 60 mg

• Fluid restriction: 1000 mL ➔ 750 mL ➔ 500 mL ➔ 0 mL

Subsequent daily study visits will evaluate the change in serum sodium over the previous 24 h, reassess volume status and then determine the dosage of tolvaptan or the limit of fluid restriction for that study day, depending on allocation. Early morning bloods will facilitate morning decision-making. Participants whose sodium level increases at the desired rate 5–8 mmol/L over 24 h will continue the same dose of their intervention. Participants whose serum sodium rise is below target will have escalation of intervention—either tolvaptan dose increase (e.g. from 7.5 to 15 mg), or reduction of fluid restriction limit (e.g. 1000 to 750 mL). Participants with decreased serum sodium despite their initial intervention will have more rapid escalation of intervention dosing (e.g. tolvaptan from 7.5 to 30 mg; or fluid restriction from 1000 to 500 ml). Participants who record a 24-h serum sodium rise > 8 mmol/L (or have required intravenous dextrose due to Na > 8 mmol above target during the 24-h period) will not receive either tolvaptan or fluid restriction the following day. Participants who achieve normalisation of serum sodium concentration (135 mmol/L or above) during the course of the trial from either arm will default to a 1500 mL ‘maintenance’ fluid restriction, unless the rise in serum Na was > 8 mmol, in which case they will have free fluid intake.

Tolvaptan doses will be administered prior to 1200 (ideally prior to 1000) to allow safe monitoring of effect during daytime hours with serum sodium measurement 6 and 12 h post dose.

**Fig. 1:**
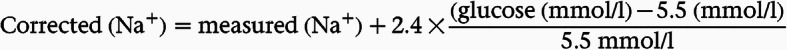
Formula for correction of sodium in the presence of hyperglycaemia, to be implemented when glucose > 10 mmol/L (> 180 mg/dL) [24]

Direct sodium measurement from venous blood gas samples will be preferred to determine outcomes and treatment decisions, unless this is not available. In the setting of glucose levels > 10 mmol/L (> 180 m/dl), a correction factor will be applied according to the formula published by Hillier and colleagues in 1999 (Fig. 1) [24], as endorsed by the European hyponatraemia guidelines [7]. This formula adds 2.4 mmol/l to the measured serum sodium concentration for every 5.5 mmol/L (100 mg/dl) incremental rise in serum glucose. For glucose levels below 10, this amounts to correction by 1 mmol/L or less, therefore for we have chosen to implement this correction for glucose > 10 mmol/L only for practical purposes.

#### Criteria for discontinuing or modifying allocated interventions {11b}

Overcorrection will be defined according to the European guidelines as an increment in plasma sodium of > 10 mmol/L in 24 h [7]. Overcorrection will be defined as a serious adverse event (SAE) and will prompt individualised management by the treating medical team in collaboration with the endocrinology consult service for decisions regarding re-lowering of plasma sodium with intravenous 5% dextrose therapy.

If the serum sodium rise has not exceeded 10 mmol/L in 24 h but is rising at a faster than desired rate, prophylactic intravenous 5% dextrose will be administered, per protocol. In the event that sodium concentration rises by > 6 mmol/L within 6 h of tolvaptan, or > 8 mmol/L within 12 h of tolvaptan administration, this will be initiated at a rate to match urine output, with increased frequency of sodium monitoring (Table 2) in accordance with safety advice issued by Otsuka Australia Pharmaceutical [28]. This infusion will be discontinued if the plasma sodium then falls back within the target range, and serial sodium monitoring will continue as standard protocol.

**Table 2:** Thresholds for intervention with intravenous dextrose 5% in the context of sodium rise following tolvaptan dose

Rise in serum sodium	Time post-tolvaptan	Action
> 6 mmol/L	Within 6 h	Commence IV dextrose 5% at rate equivalent to urine output, continue to monitor frequently and cease when sodium back within target range
> 8 mmol/L	Within 12 h	
> 10 mmol/L	Within 24 h	Aim for re-lowering with bolus hypotonic fluid

In the event that daily bloods are not available for any reason, management will revert to a 1.5-L fluid restriction for both groups while awaiting further results.

#### Strategies to improve adherence to interventions {11c}

The main challenge to adherence will be accurate implementation of fluid restriction (or lack thereof) from both a staff and patient perspective. Ward staff including doctors and nurses will be educated about the purpose and implementation of the study. Clear visual communication boards will be present at each participant’s bedside, updated daily with their fluid intake ‘prescription’, which will also be documented in the patient’s medical record. Patients will be educated about their requirements in adhering to a fluid restriction (or not) and will be encouraged to assist in recording and monitoring their fluid intake and output, which will be documented by nursing staff in the electronic patient record as per usual care practices. Any adherence challenges beyond this are expected to reflect the real-world difficulty of implementing a strict fluid restriction.

#### Relevant concomitant care permitted or prohibited during the trial {11d}

Fluid restriction will not be allowed for participants in the tolvaptan group on days that they receive tolvaptan. Tolvaptan will not be allowed for participants in the fluid restriction arm. Otherwise, management decisions will be the responsibility of the treating team. This includes the use of other treatments for hyponatraemia including diuretics, oral solute, or intravenous saline. The treating medical team will retain the authority to withdraw a participant from the trial for any reason.

In the event that a patient becomes significantly hypovolaemic (as demonstrated by development of hypotension or serial urine sodium newly < 20 mmol/L), the patient will not receive either tolvaptan or fluid restriction and may be prescribed intravenous normal 0.9% saline at the discretion of the treating team.

#### Provisions for post-trial care {30}

After completion of the 72-h trial period, patients will revert to usual care. The endocrinology unit is available at all hours on a consult basis to assist other medical teams with the management of inpatients with hyponatraemia before, during, or after the study. Patients will be contacted by phone 30 days after discharge to complete an exit questionnaire and obtain serum sodium result 30 days post discharge.

### Outcomes {12}

Primary and secondary endpoints are shown in Table 3.

**Table 3:** Outcomes

**Primary endpoint**	• Change in serum sodium concentrations over time, from day 1 to day 4
**Secondary endpoints**	• Difference between groups of area under the curve of serial direct serum sodium measurements in mmol/L • Serum sodium increment in the first 24 and 48 h • Proportion of patients normalising serum sodium (defined as serum sodium ≥ 135 mmol/L) • Length of hospital stay • Requirement for serum sodium re-lowering with enteral or IV dextrose/water and/or desmopressin o *Main safety outcome: difference between rates of above-target correction of serum sodium leading to attenuation/reversal therapy (e.g. IV dextrose administration)* • Cognitive and functional measures at 24, 48 and 72 h (or discharge if sooner) o Confusion Assessment Method Shortform (CAM-S) score o Hyponatraemia symptom questionnaire score o Timed Up and Go test score • 30-day readmission rate • Exit questionnaire treatment satisfaction score • Serum sodium concentration 30 days after discharge

### Participant timeline {13}

The participant timeline is shown in Table 4.

**Table 4:**
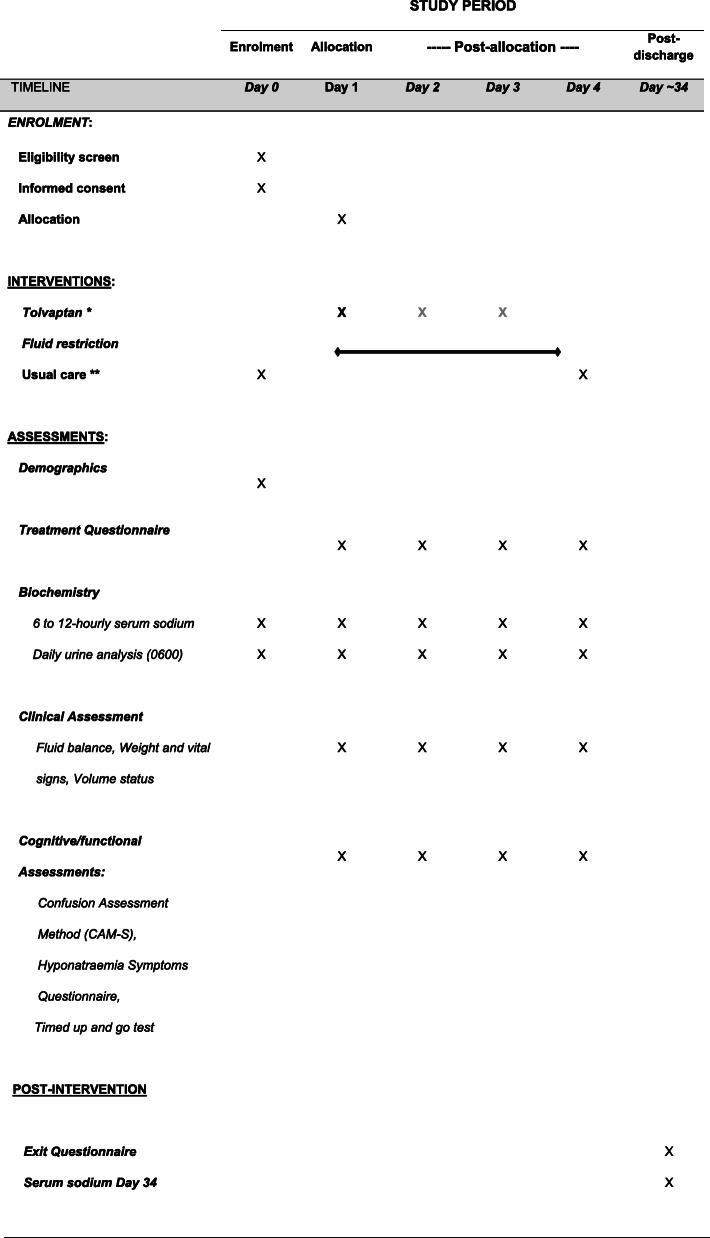
Participant timeline

*Need for subsequent tolvaptan doses on days 2 and 3 reassessed daily according to protocol (based on serum sodium increment)

**Usual care may include fluid restriction or tolvaptan (patients administered tolvaptan prior to enrolment will not be included)

### Sample size {14}

The SALT 1 trial reported a mean difference between tolvaptan and placebo groups in sodium increment at day 4, of approximately 4 mmol/L. The standard deviation (SD) in day 4 sodium concentrations was approximately 4.9 (effect size 0.8). We determined that 2 mmol/L is the minimally clinically relevant difference between tolvaptan and fluid restriction. Using the same SD, we estimated that 64 participants per group would be required to detect this difference based on a *t*-test with 80% power and a 2-sided *p*-value equal to 0.05. To allow for drop-outs including autocorrection of hyponatraemia without specific therapy, a total of 166 participants will be recruited (83 per group).

In case of unforeseen difficulty with recruitment, we have done further power estimates to determine the number of participants required to detect a 3 mmol/L and a 4 mmol/L between group difference, using the same power and significance levels (Table 5). Expected minimum recruitment is 52 participants, or 68 accounting for an additional 30% recruitment margin.

**Table 5:** Power calculations

Difference between arms	Number per group	+ 30% to allow for drop-outs and autocorrection	Total sample size
2 mmol/L	64	83	166
3 mmol/L	43	56	112
4 mmol/L	26	34	68

### Recruitment {15}

All patients admitted to a tertiary hospital in Victoria, Australia (The Austin Hospital), may be screened for potential recruitment. A daily 24-h report of all admitted patients with hyponatraemia will be extracted from the hospital electronic database (Cerner Millennium) at 0800 hours each morning. Based on this extract, the written and electronic medical records of potential participants will be screened for eligibility by study personnel. Eligible participants will be approached in person on the ward.

All of the information required for ensuring participant eligibility is obtained as part of clinical routine care of the hyponatraemic patient. However, if by oversight the clinical team has not performed a relevant blood or urine test, those will be requested by study personnel for the purposes of assessing eligibility.

## Assignment of interventions: allocation

### Sequence generation {16a}

Randomisation will be stratified according to volume state (euvolaemic or hypervolaemic) which will be defined based on the presence or absence on clinical examination of detectable dependent pitting oedema and assessment of jugular venous pressure. Randomisation will be further stratified by serum sodium concentration (Na < 123 mmol/L or Na ≥ 123 mmol/L) to ensure even distribution of biochemical severity between groups.

Four randomisation sequences based on computer-generated random numbers with varying block size (2–6) will be generated and concealed from study personnel for the duration of the study.

### Concealment mechanisms {16b}

The allocation sequence for each stratification subgroup will be placed in sequentially numbered sealed envelopes by Austin Health Clinical Trials Pharmacy staff.

### Implementation {16c}

The allocation sequences will be generated by Austin Health Clinical Trials Pharmacy staff, independent of study personnel and sequential concealment envelopes prepared. Study personnel will recruit potentially eligible patients on the ward. When a participant consents to be enrolled in the trial, they will be assigned a study number and the next sequential envelope will be accessed and opened to reveal their allocation, based on their stratification subgroup (hypervolaemic or euvolaemic, and sodium level below or above/at 123 mmol/L) to allocate the participant to one of two study arms (tolvaptan or fluid restriction). The intervention will then be communicated to the treating team, and if allocated to tolvaptan, tolvaptan will be prescribed by standard electronic prescribing methods and administered by clinical ward staff.

## Assignment of interventions: blinding

### Who will be blinded {17a}

This trial is open-label due to the nature of the interventions (fluid restriction, versus administration of tolvaptan tablet with free fluid intake). The participants’ randomised allocation will only be revealed to the investigator and participant after enrolment is complete.

### Procedure for unblinding if needed {17b}

There will be no unblinding procedure undertaken.

## Data collection and management

### Plan for assessment and collection of outcomes {18a}

Data will be collected with the use of Research Electronic Data Capture (REDCap) software hosted by Austin Health [29, 30]. This is a secure, web-based software platform designed to support data capture for research studies that is widely used in research. REDCap provides (1) an intuitive interface for validated data capture, (2) audit trails for tracking data manipulation and export procedures, (3) automated export procedures for seamless data downloads to common statistical packages and (4) procedures for data integration and interoperability with external sources. Where possible, repeat measures will be performed by a single researcher, who will be a doctor with experience in hyponatraemia management, or alternative healthcare personnel with appropriate training in the specifics of this trial. The following study measures have been built into the REDCap project application.

#### Demographics

Demographic information will be collected including name, date of birth, age, hospital record number and contact phone number (for patient or medical treatment decision maker). Patients will subsequently be identified by a study number. We will also collect information including place of residence (home versus supported care) and evaluation of baseline functional status in the form of the European Cooperative Oncology Group (ECOG) score [31] and whether the participant requires assistance to complete their activities of daily living. We will also collect a list of medical diagnoses and regular medications which will be relevant with respect to inclusion and exclusion criteria, and will inform calculation of a Charlson comorbidity index score [32] alongside a record of the presumed underlying cause of hyponatraemia.

#### Screening

A screening questionnaire will ensure that inclusion and exclusion criteria are met (see above). This requires results from a number of routine investigations to be available, e.g. serum sodium and potassium levels, serum glucose level, urine sodium and specific gravity, cortisol level, thyroid function tests and estimated glomerular filtration rate, plus clinical assessment of conscious state and volume status. If the patient is eligible for the trial and informed consent is obtained, they will be allocated to a treatment arm.

#### Treatment questionnaire

Once allocation has occurred according to the randomization process, a daily treatment questionnaire will monitor progress and assist in implementation of the titration protocol (Tables 1 and 2). Daily biochemistry and 24-h sodium increment will be recorded to determine the intervention for the following 24 h. Details of any potential adverse events, including requirement for intravenous dextrose to mitigate serum sodium rise, are recorded as escalated as required.

##### Biochemistry

Sodium measurements will be performed with simultaneous indirect urea, electrolytes and creatinine and direct measurement with venous blood gas will monitor serum sodium. In patients with serum sodium below 125 mmol/L and all patients following tolvaptan administration, bloods will be performed every 6–8 h in accordance with recommendations [7, 28]. Patient with serum sodium above 125 mmol/L being managed with a fluid restriction (not tolvaptan) will have bloods every 12 h. In the event of discrepancy between these readings, the direct sodium measurement will be preferred due to increased accuracy and faster turnaround time [33]. Daily serum osmolality will be recorded. A daily urine sample at 0600 will monitor urine sodium, osmolality and specific gravity. Daily monitoring of liver function will be performed in tolvaptan recipients for safety given the small risk of liver damage identified in longer-term trials of high-dose tolvaptan used for other indications [23].

##### Clinical assessment

Daily weight, vital signs and fluid balance (intake and output) will be obtained from the routine electronic medical record. In addition, daily clinical assessment of volume status will be performed to ensure ongoing eligibility and safety.

#### Cognitive/functional assessments

##### Confusion Assessment Method Shortform (CAM-S)

The CAM-S is a widely-used, validated tool for identifying delirium and scoring its severity [34]. It consists of four domains assessing core features of delirium, each assigned a score between 0 and 2 to grade severity. The domains are as follows: acute onset and fluctuating course, inattention, disorganized thinking and altered level of consciousness. The CAM-S is scored by the assessor with a minimum score of 0 and maximum of 7. It will be assessed at baseline (day 1) and repeated 24, 48 and 72 h after commencing the intervention arm (days 2–4) to quantify progress. The CAM has a sensitivity of 94–100%, specificity of 90–95% in detecting delirium and high inter-rater reliability [35, 36].

##### Hyponatraemia symptom questionnaire

A hyponatraemia symptom questionnaire has been devised comprised of two sections. The first is a visual analogue scale scored from 1 to 10 that asks participants to rate their headache, nausea and unsteadiness respectively. Participants will indicate the visual location along a bar from 0 ‘none at all’ to 10 ‘worst you can imagine’. The RedCAP software will calculate a score from 1 to 10 to two decimal places based on the placement of the slider by the participant for each of the three potential symptoms. The second component is the Hyponatraemia Disease-Specific Survey (HDSS), adapted from pre-specified but exploratory symptom evaluation in the SALT-2 tolvaptan trial [13]. There are some similarities to the SF-12 Short Form Health Survey which evaluated wellbeing in both SALT trials; however, that tool is not hyponatraemia-specific [11, 37]. In the HDSS, participants are asked to rate their health over the past day (excellent, very good, good, fair or poor). They are then asked how much their thinking ability has been limited, specifically: Concentrating, Calculating, Language and Memory ability (not at all, slightly, moderately, quite a bit, extremely), which will be scored from 0 to 4 to derive a ‘mental component summary score’ from 0 to 16 (higher score indicating higher level of self-identified limitation). Participants then rate how their strength and coordination has been limited, specifically Endurance, Strength, Gross coordination and Fine coordination (not at all, slightly, moderately, quite a bit or extremely), which are also attributed a number to derive a physical component score 0–16. Additional questions include self-perception of current sodium level without prompting by knowledge of their laboratory test (very low, a little low, normal). Participants are also asked to rate their level of thirst sensation (not thirsty, a little thirsty, normal thirst, extra thirst, very thirsty) and to provide an assessment of their progress ‘Overall, compared to before you came to hospital, how much better do you feel?’ (much better, somewhat better, about the same, somewhat worse or much worse).

##### Timed Up and Go test

The Timed Up and Go test (TUG) is a widely used method to assess function and potentially falls risk that has been endorsed by the American and British Geriatric Societies [38]. Participants are instructed to rise from a standard armchair, walk to a marker 3 m away, turn, walk back and sit down again [39]. It is a marker of physical function, with expected time of completion < 12 s in older adults [40, 41]. It has high test-retest validity [42].

#### Exit questionnaire

An exit questionnaire will be administered by telephone approximately 30 days post discharge. Data will be recorded including length of stay, time to normalization of serum sodium (if achieved beyond the intervention timeframe) and whether re-admission to hospital has occurred. Participants’ wellbeing will be evaluated with the question: ‘Overall, compared to before you came to hospital, how much better do you feel’ (much better, somewhat better, about the same, somewhat worse, much worse). Participant satisfaction with treatment will also be assessed: ‘Overall, how happy were you with your hospital treatment for hyponatraemia (low sodium)?’ (very happy, somewhat happy, neutral somewhat unhappy, very unhappy). The result of serum sodium measurement 30 days post discharge obtained as part of routine care will be collected.

### Plans to promote participant retention and complete follow-up {18b}

The short duration of the 3-day intervention and the hospital inpatient setting are factors that are expected to promote participant retention. Participants are free to withdraw from the trial at any time and will be made aware that this will not affect their routine medical care. Analysis will be conducted according to an intention-to-treat principle. In the event that participants experience an above-target rise in serum sodium >10mml/L and exit the study to receive re-lowering intravenous dextrose therapy, the primary endpoint will not be reflective of the effect of the intervention. However, it will still reflect the summative effect of pursing that therapy first-line with appropriate safeguards in place. It will therefore still be useful in assessing whether safe correction of sodium is achieved in a timely manner.

### Data management {19}

Data will be collected via direct entry into the Research Electronic Data Capture (REDCap) software database via an application on a password-protected tablet device (Apple iPad) at the participant bedside. This reduces ‘double handling’ of data. Numerical input fields will have range checks for data values to minimize the chance of data entry error. The data will be stored in the secure cloud-based Austin Health REDCap server, with de-identified secure backup copies made weekly.

### Confidentiality {27}

Personal information will be collected only if consent to participate is obtained. Details including name, date of birth, hospital record number and telephone number will be collected to allow the day 34 exit interview to take place. This data will be stored on the secure cloud-based REDCap platform on an Austin Health server. Only authorized study personnel will be granted password-protected access. Participants will be allocated a sequential study identification number for use during the study. Only de-identified information will be published or reported. Electronic and paper study files will be kept for 15 years at which point they will be securely destroyed. Data security will be the responsibility of the primary investigator.

### Plans for collection, laboratory evaluation and storage of biological specimens for genetic or molecular analysis in this trial/future use {33}

Blood and urine biochemistry results will be obtained from samples taken as part of routine hospital care. A sub-study to evaluate serum copeptin before and after intervention to determine if it may predict response to either tolvaptan or fluid restriction is planned, which will require frozen storage of serum samples to be processed at a later date.

## Statistical methods

### Statistical methods for primary and secondary outcomes {20a}

For descriptive data, we will report either mean ± SD or median [IQR] depending on the distribution of the data. Change in serum sodium from baseline (day 1 0600 hours) to day 4 0600 hours (or day of discharge if earlier) will be compared between treatment groups. We will also compare the change in average daily area under the curve (AUC) for the serum sodium concentration from baseline to day 4, in line with analysis performed in the SALT trials [11]. A mixed-effects model based on restricted maximum likelihood (REML) will be used to account for both within-subject variation over time and between-subject variation to assess the treatment effect, namely the mean adjusted difference (MAD) between the control and the tolvaptan group surrounded by a profiled 95% confidence interval. Analysis will be by the intention-to-treat principle (ITT) so that data from participants who withdraw or violate the protocol will be included in their assigned treatment group. We will also perform a per-protocol sensitivity analysis. Plans for the statistical analysis and reporting of each secondary outcome will be decided upon by investigators with access to pooled data.

### Interim analyses {21b}

Interim analyses are not planned.

### Methods for additional analyses (e.g. subgroup analyses) {20b}

Subgroup analyses will be performed in a similar mixed-effects model, with respect to effect of volume status (hypervolaemic vs euvolaemic), serum sodium level (mmol/L), urine osmolality (mOsm/kg), urine/plasma osmolality ratio and urine sodium (mmol/L).

### Methods in analysis to handle protocol non-adherence and any statistical methods to handle missing data {20c}

Assuming missingness at random (MAR), this is handled by the likelihood-based mixed effects model. In the event of non-missingness at random (NMAR), joint modelling multiple imputation is available as an alternative. As a sensitivity analysis, a per protocol (PP) analysis is performed, including only patients who adhered to the protocol and completed the trial.

### Plans to give access to the full protocol, participant-level data and statistical code {31c}

The full study protocol will be available by request. We will aim to publish data in aggregate only; however, some scholarly journals require de-identified individual-level data to be shared so that statistical analyses can be verified on request. In the event of reasonable request, individual-level data will be de-identified prior to sharing. Statistical code will be available in supplementary material with the final published manuscript.

## Oversight and monitoring

### Composition of the coordinating centre and trial steering committee {5d}

The trial steering committee comprised of the investigator group will meet fortnightly to oversee the execution of the trial. The day-to-day running of the trial will be the responsibility of the primary investigator who will be involved in recruitment, assessment and implementation. The trial will also be overseen by a statistician and the Austin Health Endocrinology specialist department providing additional expert input if required, in addition to the Austin Health Ethics Committee and independent Data and Safety and Monitoring Board for the trial.

### Composition of the data monitoring committee, its role and reporting structure {21a}

An independent Data and Safety Monitoring Board (DSMB) will be appointed. This will be chaired by an independent senior endocrinologist and researcher with expertise in management of hyponatraemia and comprise 2 additional independent expert clinicians. The DSMB members will be independent of Austin Health, the Sponsor (University of Melbourne) and the funding source (Otsuka Australia Pharmaceutical) and will be free from competing interests. The DSMB charter is available on request. The DSMB will receive 3-monthly progress reports as well as a report on all safety adverse events (SAEs) and have the power to recommend modification or discontinuation of the trial if concerns are identified.

### Adverse event reporting and harms {22}

During the study, from the time the informed consent is signed by the patient until 28 calendar days following the study participation, safety information will be collected by means of completing case report forms and adverse event forms.

Adverse events will be reported to the trial Data Safety and Monitoring Board who will determine whether any protocol amendment or discontinuation is warranted.

In addition, Serious Adverse Events and Product Quality Complaints relating to tolvaptan will be reported to the sponsor University of Melbourne and the funding source Otsuka Australia Pharmaceutical within 24 h of first awareness of the event for submission to the Global Pharmacovigilance database. The collection period of safety information is from the first use of tolvaptan until 28 calendar days after discontinuation. De-identified participant data (for example, referring to patients using their sex and age or date of birth, but not their names or contact details) may be sent to Otsuka. Serious adverse events and all other safety information will be included in the Final Study Report to Otsuka at the end of the study.

As stated, the sponsor, University of Melbourne, and the funding source, Otsuka Australia Pharmaceutical, have no role in the study design, data analysis or manuscript preparation.

### Frequency and plans for auditing trial conduct {23}

Reports every 3 months to the independent Data Safety and Monitoring Board will include audit of trial conduct.

### Plans for communicating important protocol amendments to relevant parties (e.g. trial participants, ethical committees) {25}

Protocol modifications will require external authorisation via amendments to the existing ethics approval by the Austin Health Human Research Ethics Committee. Protocol modification will only be made with the approval of the investigation team and will be communicated to the Data Safety and Monitoring board and all study personnel. Where necessary, participants will be notified and will sign an updated participant information and consent form. Updates will be communicated to any journal in which the trial methods appear, and the registration with the Australia and New Zealand Clinical Trial Registry will be maintained with current information.

### Dissemination plans {31a}

On study completion, all participants will be sent a letter summarising the study results and inviting them to contact the research team with any questions. The trial will be published in an international peer-reviewed journal. Abstracts will also be submitted to relevant national and international endocrine and internal medicine scientific meetings.

## Discussion

Tolvaptan has the potential to have great utility in the treatment of patients with moderate-to-profound hyponatraemia, but its uptake has been limited by lack of evidence for such patients. In additional, there are concerns regarding safety, level of benefit and potential cost. The absence of evidence is reflected in the divergent recommendations in current hyponatraemia guidelines [4, 7].

Hyponatraemia is a common clinical management problem, and until recently, the complexity and need for individualised therapy may have contributed to a lack of reliable evidence in this field.

Recent research has interrogated traditional management to provide an evidence base for fluid restriction in trial conditions and has reported limitations with this first line approach [26, 27]. Our study will provide a randomised comparison of tolvaptan and fluid restriction, which, despite observational trials and growing clinical experience, is yet to be formally evaluated [14–17].

Limitations of this study include its open-label design, necessary use of an unvalidated hyponatraemia symptom questionnaire, and single-centre operation. Another limitation common to all trials in hyponatraemia in hospital is that a proportion of patients with SIAD may improve spontaneously without, or despite, intervention, due to resolution of the underlying cause of non-osmotic vasopressin release. We are mitigating this issue by delaying recruitment at least 24–48 h after hyponatraemia is identified. Strengths of this trial include its prospective, randomised design, and a pre-specified protocol to reassess and titrate interventions daily, in keeping with clinical practice. Another strength is use of the increasingly recommended lower dose 7.5 mg of tolvaptan that has not been assessed in previous randomised trials.

Tolvaptan is known to be efficacious in raising sodium concentration, and we therefore seek to quantify its benefit and establish its safety in raising serum sodium concentrations in moderate to profound hyponatraemia in replicable conditions, with protocolised dosing and monitoring. Any benefit in cognitive and functional outcomes, reduction in length of hospital stay or reassuring safety information would provide additional rationale for tolvaptan use, which may be required to overcome the immediate cost advantage and accessibility of fluid restriction. We hope to clarify the utility of tolvaptan in clinical practice and inform international guidelines with high-quality data, and provide an evidence-based framework for tolvaptan initiation and monitoring.

## Trial status

Latest protocol version 16, 16 April 2021. Recruitment began in May 2021 and is scheduled to be completed by December 2022.

## Supplementary Information

**Additional file 1: Appendix 1.** Austin Health Human Research Ethics Committee Approval Letter

**Additional file 2: Appendix 2.** Funding agreement with Otsuka Australia Pharmaceutical

**Additional file 3: Appendix 3.** Tolvaptan (Samsca) product information

**Additional file 4: Appendix 4**

## Abbreviations

AUC: Area under the curve
AVP: Arginine vasopressin, also known as antidiuretic hormone (ADH)
BMI: Body mass index, i.e. weight (in kilogrammes) divided by height (in metres) squared
CAM-S: Confusion Assessment Method, Short form
CKD: Chronic kidney disease
DSMB: Data Safety and Monitoring Board
ECOG: Eastern Co-operative Oncology Group Score
eGFR: Estimated glomerular filtration rate
GCS: Glasgow Coma Scale
HDSS: Hyponatraemia Disease-Specific Survey
Na: Sodium
RedCAP: Research Electronic Data Capture
SAE: Serious adverse event
SIAD: Syndrome of inappropriate diuresis, also known as syndrome of inappropriate antidiuretic hormone (SIADH)
TSH: Thyroid stimulating hormone
TUG: Timed Up and Go test
UEC: Urea, electrolytes and creatinine
VBG: Venous blood gas
