# Supplementary material for: Tolvaptan versus fluid restriction in acutely hospitalised patients with moderate-profound hyponatraemia (TVFR-HypoNa): design and implementation of an open-label randomised trial
Source: Trials. 2022 Apr 21;23:335. doi: 10.1186/s13063-022-06237-5 (PMC9028077; doi:10.1186/s13063-022-06237-5)
Supplement: Supplementary file 2 — Additional file 2: Appendix 2. Funding agreement with Otsuka Australia Pharmaceutical [file 13063_2022_6237_MOESM2_ESM.pdf]

Otsuka

Otsuka-people creating new products for better health worldwide

Otsuka Australia Pharmaceutical Pty Ltd
ABN: 20 601 768 754
Suite 2.03, Level 2, 9 Help Street, Chatswood 2067
Telephone: +61 (0) 2 8021 9825
Facsimile: +61 (0) 2 8021 9963

To whom it may concern,

Otsuka has agreed to provide $75,000 in funding to Prof Mathis Grossmann in the form of a research grant to support his investigator-initiated study of tolvaptan versus fluid restriction (IIT 156-419-00250). The schedule of payments is as described in Appendix II of the document 'Agreement for Support of an Investigator-Initiated Study'. Otsuka provides this support without any intention to induce or reward the past, present or future prescribing, supply, purchasing or recommendation of Otsuka products.

Yours faithfully,

F. Castaldi

Francesco Castaldi
Medical Manager
Otsuka Australia Pharmaceutical Pty Ltd
Suite 2.03, Level 2, 9 Help Street, Chatswood 2067
E: Francesco.Castaldi@au.otsuka.com
M: 0419 310 896
Fax: 02 8021 9963
