# Supplementary material for: Tolvaptan versus fluid restriction in acutely hospitalised patients with moderate-profound hyponatraemia (TVFR-HypoNa): design and implementation of an open-label randomised trial
Source: Trials. 2022 Apr 21;23:335. doi: 10.1186/s13063-022-06237-5 (PMC9028077; doi:10.1186/s13063-022-06237-5)
Supplement: Supplementary file 3 — Additional file 3: Appendix 3. Tolvaptan (Samsca) product information [file 13063_2022_6237_MOESM3_ESM.pdf]

AUSTRALIAN PRODUCT INFORMATION
SAMSCA® (TOLVAPTAN) TABLETS

1 NAME OF THE MEDICINE

Tolvaptan

2 QUALITATIVE AND QUANTITATIVE COMPOSITION

SAMSCA tablets for oral use contain 15 mg or 30 mg of tolvaptan.

Excipients with known effect: sugars as lactose.

For the full list of excipients, see Section 6.1 LIST OF EXCIPIENTS.

3 PHARMACEUTICAL FORM

SAMSCA is supplied as 15 mg and 30 mg tolvaptan tablets.

The 15 mg tablet is blue, triangular, shallow-convex, debossed with “OTSUKA” and “15” on one side.

The 30 mg tablet is blue, round, shallow-convex, debossed with “OTSUKA” and “30” on one side.

4 CLINICAL PARTICULARS

4.1 THERAPEUTIC INDICATIONS

SAMSCA is indicated for the treatment of clinically significant hypervolemic or euvolemic hyponatremia (serum sodium less than 125 mmol/L, or less marked hyponatremia that is symptomatic and has resisted correction with fluid restriction) including patients with heart failure and Syndrome of Inappropriate Antidiuretic Hormone (SIADH).

Important Limitations

Patients requiring intervention to raise serum sodium urgently to prevent or to treat serious neurological symptoms should not be treated with SAMSCA. It has not been established that raising serum sodium with SAMSCA provides a symptomatic benefit to patients.

4.2 DOSE AND METHOD OF ADMINISTRATION

Due to the need for a dose titration phase with close monitoring of serum sodium and volume status, **treatment with SAMSCA should be initiated and re-initiated in hospital.**

Treatment with SAMSCA should be initiated at a dose of 15 mg, orally, once daily. The dose may be increased to a maximum of 60 mg once daily as tolerated to achieve the desired level

of serum sodium. Increase from the starting dose should be done incrementally (first to 30 mg and then to 60 mg if required) at intervals ≥ 24 hours. During titration, patients should be monitored for serum sodium and volume status.

For patients with an appropriate increase in serum sodium, the underlying disease and serum sodium levels should be monitored at regular intervals to evaluate further need of SAMSCA treatment. Do not administer SAMSCA for more than 30 days to minimise the risk of liver injury (see Section 4.4 SPECIAL WARNINGS AND PRECAUTIONS FOR USE).

SAMSCA tablets should be taken orally, preferably in the morning, without regard to meals. Tablets should be swallowed without chewing with a glass of water.

SAMSCA tablets should not be taken with grapefruit juice.

Patient Monitoring

1. In patients receiving SAMSCA who develop too rapid a rise in serum sodium, treatment with SAMSCA should be discontinued or interrupted and administration of hypotonic fluids should be considered. Fluid restriction during the first 24 hours of therapy with SAMSCA may increase the likelihood of overly-rapid correction of serum sodium, and should generally be avoided.

2. Hyperkalaemia or Drugs that Increase Serum Potassium.

Serum potassium levels should be monitored after initiation of SAMSCA treatment in patients with a serum potassium > 5 mmol/L as well as those who are receiving drugs known to increase serum potassium levels.

3. Drug withdrawal.

Following discontinuation of SAMSCA, patients should be advised to resume fluid restriction and should be monitored for changes in serum sodium and volume status.

4.3 CONTRAINDICATIONS

SAMSCA is contraindicated in;

- Patients hypersensitive to the active substance, benzazepine derivatives or any of the excipients.
- Patients who are unable to sense or appropriately respond to thirst.
- Patients suffering from hypovolaemic hyponatraemia.
- Patients with anuria.
- Urgent need to raise serum sodium acutely. SAMSCA has not been studied in a setting of urgent need to raise serum sodium acutely.
- Ketoconazole and Other Strong CYP 3A Inhibitors. See Section 4.5 INTERACTIONS WITH OTHER MEDICINES AND OTHER FORMS OF INTERACTION.

4.4 SPECIAL WARNINGS AND PRECAUTIONS FOR USE

Heart failure

Efficacy of SAMSCA for the treatment of heart failure has not been established.

Hypothyroidism and Hypoadrenalism

Patients should be screened and treated for hypothyroidism and hypoadrenalism before treatment with SAMSCA.

Liver injury

Drug induced liver injury has been observed in clinical trials investigating a different indication with long-term use of SAMSCA at higher doses than for the approved indication. In a placebo-controlled and open label extension study of chronically administered tolvaptan in patients with autosomal dominant polycystic kidney disease (ADPKD), clinically significant increases (greater than 3 x Upper Limit of Normal) in serum alanine aminotransferase (ALT), along with clinically significant increases (greater than 2 x Upper Limit of Normal) in serum total bilirubin were observed in 3 patients treated with SAMSCA. In addition, an increased incidence of ALT greater than three times the upper limit of normal was observed in patients treated with SAMSCA [42/958 or 4.4%] compared to those receiving placebo [5/484 or 1.0%]. Cases of serious liver injury were generally observed starting 3 months after initiation of tolvaptan although elevations of ALT occurred prior to 3 months. These findings may suggest that SAMSCA can cause irreversible and potentially fatal liver injury.

In postmarketing experience with tolvaptan in ADPKD, acute liver failure requiring liver transplantation has been reported.

Liver function tests should be promptly performed in patients taking SAMSCA who report symptoms that may indicate liver injury, including fatigue, anorexia, right upper abdominal discomfort, dark urine or jaundice. If liver injury is suspected, SAMSCA should be promptly discontinued, appropriate treatment should be instituted, and investigations should be performed to determine the probable cause. SAMSCA should not be re-initiated in patients unless the cause for the observed liver injury is definitively established to be unrelated to treatment with SAMSCA.

Limit duration of therapy with SAMSCA to 30 days. Avoid use in patients with underlying liver disease, including cirrhosis, because the ability to recover from liver injury may be impaired.

Too rapid increases in serum sodium

Patients should be monitored frequently during initiation and after each titration for serum sodium and volume status to avoid any adverse neurologic outcomes that may result from rapid increases (e.g., >12 mmol/L/24 hr) in serum sodium. Too rapid correction of hyponatraemia can cause osmotic demyelination which may result in dysarthria, mutism, dysphagia, lethargy, affective changes, spastic quadriparesis, seizures, coma and death.

Co-administration of SAMSCA with other treatments for hyponatraemia, such as hypertonic saline or other therapies that may increase serum sodium levels, is not recommended, particularly during initiation or in patients with fluctuating serum sodium. Correction of hyponatremia should be slower particularly in patients suffering from severe malnutrition, alcoholism, SIADH, hypoxia or advanced liver disease.

In patients receiving tolvaptan who develop too rapid a rise in serum sodium, discontinue or interrupt treatment with tolvaptan and consider administration or ingestion of hypotonic fluids as appropriate.

Hypernatremia

Potent aquaresis (free water clearance), may induce hypernatremia which can lead to adverse neurologic events. If serum sodium increases above normal range, tolvaptan dose should be adjusted, serum sodium should be carefully monitored and appropriate measures should be taken if necessary.

Dehydration and hypovolaemia

Patients receiving SAMSCA should have access to water and should continue ingestion of fluid in response to thirst.

SAMSCA therapy induces copious aquaresis, which is normally partially offset by fluid intake. Dehydration and hypovolaemia can occur, especially in potentially volume-depleted patients receiving diuretics or those who are fluid restricted.

In patients treated with SAMSCA who develop medically significant signs or symptoms of hypovolaemia, treatment with SAMSCA should be discontinued or interrupted. In such patients the fluid balance, electrolyte levels and vital signs should be carefully managed. Fluid restriction during therapy with SAMSCA may increase the risk of dehydration and hypovolaemia.

Hyperkalaemia or drugs that increase serum potassium

Treatment with SAMSCA is associated with an acute reduction of the extracellular fluid volume which could result in increased serum potassium. Serum potassium levels should be monitored after initiation of SAMSCA treatment in patients with a serum potassium > 5 mmol/L as well as those who are receiving drugs known to increase serum potassium levels.

Urinary outflow obstruction

Urinary output must be secured. Patients with partial obstruction of urinary outflow have an increased risk of developing acute retention. Patients with urinary outflow obstructions must be monitored to ensure urinary output is achieved.

Lactose and galactose intolerance

SAMSCA contains lactose as an excipient. Patients with rare hereditary problems of galactose intolerance, the Lapp lactase deficiency or glucose-galactose malabsorption should not take this medicine.

Use in hepatic impairment

Moderate and severe hepatic impairment do not affect exposure to SAMSCA to a clinically relevant extent. Avoid use of tolvaptan in patients with underlying liver disease.

Use in renal impairment

Exposure and response to SAMSCA are similar in patients with a creatinine clearance 10 79 mL/min and in patients without renal impairment. No dose adjustment is necessary. Exposure and response to SAMSCA in patients with a creatinine clearance < 10 mL/min or in patients on

chronic dialysis have not been studied. SAMSCA is contraindicated in anuric patients. No benefit can be expected in patients who are anuric.

Use in the elderly

Of the total number of hyponatraemic subjects treated with SAMSCA in clinical studies, 42% were 65 and over, while 19% were 75 and over. No overall differences in safety or effectiveness were observed between these subjects and younger subjects, and other reported clinical experience has not identified differences in responses between the elderly and younger patients, but greater sensitivity of some older individuals cannot be ruled out.

Increasing age has no effect on tolvaptan plasma concentrations.

Paediatric use

Safety and effectiveness of SAMSCA in pediatric patients have not been established.

Effects on laboratory tests

No data available.

4.5 INTERACTIONS WITH OTHER MEDICINES AND OTHER FORMS OF INTERACTIONS

Ketoconazole and Other Strong CYP 3A Inhibitors

Use of Ketoconazole and Other strong CYP 34A inhibitors with SAMSCA is contraindicated (See Section 4.3 CONTRAINDICATIONS). SAMSCA is metabolized primarily by CYP 3A. Ketoconazole is a strong inhibitor of CYP 3A and also an inhibitor of P-glycoprotein. Co-administration of SAMSCA and ketoconazole 200 mg daily results in a 5-fold increase in exposure to SAMSCA. Co-administration of SAMSCA with 400 mg ketoconazole daily or with other strong CYP 3A inhibitors (e.g., clarithromycin, itraconazole, telithromycin, saquinavir, nelfinavir, ritonavir and nefazodone) at the highest labeled dose would be expected to cause an even greater increase in SAMSCA exposure. Thus, SAMSCA and strong CYP 3A inhibitors should not be co-administered.

Moderate CYP 3A Inhibitors

The impact of moderate CYP 3A inhibitors (e.g., erythromycin, fluconazole, aprepitant, diltiazem and verapamil) on the exposure to co-administered SAMSCA has not been assessed. A substantial increase in the exposure to SAMSCA would be expected when SAMSCA is co-administered with moderate CYP 3A inhibitors. Co-administration of SAMSCA with moderate CYP3A inhibitors should therefore generally be avoided.

Grapefruit Juice

SAMSCA should not be taken with grapefruit juice.

Rifampin and Other CYP 3A Inducers

Rifampin is an inducer of CYP 3A and P-glycoprotein. Co-administration of rifampin and SAMSCA reduces exposure to tolvaptan by 85%. Therefore, the expected clinical effects of SAMSCA in the presence of rifampin and other inducers (e.g., rifabutin, rifapentin, barbiturates, phenytoin, carbamazepine and St. John's Wort) may not be observed at the usual dose levels of SAMSCA. The dose of SAMSCA may have to be increased.

P-glycoprotein (P-gp) Inhibitors

Tolvaptan is a substrate for P-glycoprotein. Reduction in the dose of SAMSCA may be required in patients concomitantly treated with P-gp inhibitors, such as e.g., ciclosporin, based on clinical response.

Lovastatin, Warfarin, Amiodarone, Frusemide, and Hydrochlorothiazide

Co-administration of SAMSCA does not appear to alter the pharmacokinetics of lovastatin, warfarin, frusemide, hydrochlorothiazide, or amiodarone (or its active metabolite, desethylamiodarone) to a clinically significant degree.

Digoxin

Steady state digoxin concentrations have been increased (1.3-fold increase in maximum observed plasma concentration [Cmax] and 1.2-fold increase in area under the plasma concentration-time curve over the dosing interval [AUCτ]) when co administered with multiple once daily 60 mg doses of SAMSCA. Patients receiving digoxin should therefore be evaluated for excessive digoxin effects when treated with SAMSCA.

Co-administration with Hypertonic Saline

There is no experience with concomitant use of SAMSCA and hypertonic saline. Concomitant use with hypertonic saline is not recommended.

Co-administration with Vasopressin Analogs

In addition to its renal aquaretic effect, SAMSCA is capable of blocking vascular vasopressin V2 receptors involved in the release of coagulation factors (e.g. von Willebrand's factor) from endothelial cells. Therefore, the effect of vasopressin analogs such as desmopressin (DDAVP) may be attenuated in patients using such analogs to prevent or control bleeding when co-administered with SAMSCA.

4.6 FERTILITY, PREGNANCY AND LACTATION

Effects on fertility

Fertility was unaffected in male and female rats given tolvaptan at oral doses up to 1000 mg/kg/day (yielding 1.3-times in males and 3.4-times in females the AUC in patients at the maximum recommended human dose [MRHD] of 60 mg per day). However, oestrus cycles were altered in rats at oral doses ≥300 mg/kg/day (2.5-times the clinical AUC at the MRHD).

Use in pregnancy

Category D

There are no adequate and well controlled studies of SAMSCA use in pregnant women. SAMSCA should not be used during pregnancy unless the potential benefit clearly justifies the potential risk to the fetus.

Tolvaptan and/or its metabolites were shown to cross the placenta in rats and rabbits. In rats treated with tolvaptan during organogenesis, reduced fetal weights and delayed fetal ossification occurred at an oral dose of 1000 mg/kg/day (yielding 12-times the clinical AUC at the MHRD. In rabbits, there were abortions at oral doses ≥300 mg/kg/day (≥ 0.8-times the clinical AUC at the MRHD). At 1000 mg/kg/day (about 5-times the clinical AUC at the MRHD), there were increased rates of embryo-fetal death, fetal microphthalmia, open eyelids, cleft palate, brachymelia and skeletal malformations. These adverse effects on embryofetal development were observed in conjunction with maternal toxicity (reduced maternal body weight gain and food consumption) although a direct effect of the drug cannot be excluded.

The effect of SAMSCA on labour and delivery in humans is unknown.

Use in lactation

It is not known whether tolvaptan is excreted into human milk. Studies in rats have shown excretion of tolvaptan and/or its metabolites in breast milk at high levels. Breast feeding should be discontinued when receiving tolvaptan.

4.7 EFFECTS ON ABILITY TO DRIVE AND USE MACHINES

When driving vehicles or using machines it should be taken into account that occasionally dizziness, asthenia or syncope may occur.

4.8 ADVERSE EFFECTS (UNDESIRABLE EFFECTS)

In multiple-dose, placebo-controlled trials, 607 hyponatraemic patients (serum sodium < 135 mmol/L) were treated with SAMSCA. The mean age of these patients was 62 years; 70% of patients were male and 82% were Caucasian. One hundred eighty nine (189) SAMSCA - treated patients had a serum sodium < 130 mmol/L, and 52 patients had a serum sodium < 125 mmol/L. Hyponatraemia was attributed to cirrhosis in 17% of patients, heart failure in 68% and SIADH/other in 16%. Of these patients, 223 were treated with the recommended dose titration (15 mg titrated to 60 mg as needed to raise serum sodium).

Overall, over 4,000 patients have been treated with oral doses of SAMSCA in open-label or placebo-controlled clinical trials. Approximately 650 of these patients had hyponatraemia; approximately 219 of these hyponatraemic patients were treated with SAMSCA for 6 months or more.

The most common adverse reactions (incidence ≥ 5% more than placebo) seen in two 30-day, double-blind, placebo-controlled hyponatraemia trials in which SAMSCA was administered in titrated doses (15 mg to 60 mg once daily) were thirst, dry mouth, asthenia, constipation, pollakiuria or polyuria and hyperglycemia. In these trials, 10% (23/223) of SAMSCA -treated patients discontinued treatment because of an adverse event, compared to 12% (26/220) of

placebo-treated patients; no adverse reaction resulting in discontinuation of trial medication occurred at an incidence of > 1% in tolvaptan-treated patients.

Table 1 lists the adverse reactions reported in SAMSCA -treated patients with hyponatraemia (serum sodium < 135 mmol/L) and at a rate at least 2% greater than placebo-treated patients in two 30-day, double-blind, placebo-controlled trials. In these studies, 223 patients were exposed to tolvaptan (starting dose 15 mg, titrated to 30 and 60 mg as needed to raise serum sodium). Adverse events resulting in death in these trials were 6% in SAMSCA -treated-patients and 6% in placebo-treated patients.

Table 1. Adverse Reactions (> 2% more than placebo) in SAMSCA -Treated Patients in Double-Blind, Placebo-Controlled Hyponatraemia Trials

System Organ Class<br>MedDRA Preferred Term	SAMSCA<br>15 mg/day to 60 mg/day<br>(N = 223)<br>n (%)	Placebo<br><br>(N = 220)<br>n (%)
**Gastrointestinal Disorders**		
Dry mouth	28 (13)	9 (4)
Constipation	16 (7)	4 (2)
**General Disorders and Administration Site Conditions**		
Thirst[a]	35 (16)	11 (5)
Asthenia	19 (9)	9 (4)
Pyrexia	9 (4)	2 (1)
**Metabolism and Nutrition Disorders**		
Hyperglycaemia[b]	14 (6)	2 (1)
Anorexia[c]	8 (4)	2 (1)
**Renal and Urinary Disorders**		
Pollakiuria or polyuria[d]	25 (11)	7 (3)

The following terms are subsumed under the referenced ADR in Table 2:
[a] polydipsia; [b] diabetes mellitus; [c] decreased appetite; [d] urine output increased, micturition urgency, nocturia

In a subgroup of patients with hyponatraemia (N = 475, serum sodium < 135 mmol/L) enrolled in a double-blind, placebo-controlled trial (mean duration of treatment was 9 months) of patients with worsening heart failure, the following adverse reactions occurred in tolvaptan-treated patients at a rate at least 2% greater than placebo: mortality (42% tolvaptan, 38% placebo), nausea (21% tolvaptan, 16% placebo), thirst (12% tolvaptan, 2% placebo), dry mouth (7% tolvaptan, 2% placebo) and polyuria or pollakiuria (4% tolvaptan, 1% placebo).

The following adverse reactions occurred in < 2% of hyponatraemic patients treated with SAMSCA and at a rate greater than placebo in double-blind placebo-controlled trials (N = 607 tolvaptan; N = 518 placebo) or in < 2% of patients in an uncontrolled trial of patients with hyponatraemia (N = 111) and are not mentioned elsewhere in the label.

Blood and Lymphatic System Disorders: Disseminated intravascular coagulation

Cardiac Disorders: Intracardiac thrombus, ventricular fibrillation

Investigations: Prothrombin time prolonged

Gastrointestinal Disorders: Ischemic colitis

Metabolism and Nutrition Disorders: Diabetic ketoacidosis

Musculoskeletal and Connective Tissue Disorders: Rhabdomyolysis

Nervous System: Cerebrovascular accident
Renal and Urinary Disorders: Urethral hemorrhage
Reproductive System and Breast Disorders (female): Vaginal hemorrhage
Respiratory, Thoracic, and Mediastinal Disorders: Pulmonary embolism, respiratory failure
Vascular disorder: Deep vein thrombosis

Hyperkalaemia also occurred commonly (≥2% and <10%). See Section 4.4 SPECIAL WARNINGS AND PRECAUTIONS FOR USE – Hyperkalaemia or Drugs that Increase Serum Potassium.

Gastrointestinal Bleeding in Patients with Cirrhosis

In patients with cirrhosis treated with tolvaptan in the hyponatremia trials, gastrointestinal bleeding was reported in 6 out of 63 (10%) tolvaptan-treated patients and 1 out of 57 (2%) placebo treated patients.

Post Marketing Experience

The following adverse reaction has been reported spontaneously during the post-marketing period. The exact incidence of the spontaneously reported adverse reactions is unknown.

- Osmotic demyelination syndrome (central pontine myelinolysis)
- Hypernatremia
- Anaphylaxis

Reporting suspected adverse reactions

Reporting suspected adverse reactions after registration of the medicinal product is important. It allows continued monitoring of the benefit-risk balance of the medicinal product. Healthcare professionals are asked to report any suspected adverse reactions at http://www.tga.gov.au/reporting-problems.

4.9 OVERDOSE

Contact the Poisons Information Centre on telephone 13 11 26 for advice on management of overdose.

Single oral doses up to 480 mg and multiple doses up to 300 mg of SAMSCA, taken once daily for 5 days have been well tolerated in studies in healthy subjects.

There is no specific antidote for SAMSCA overdose. The signs and symptoms of an acute overdose can be anticipated to be those of excessive pharmacologic effect: a rise in serum sodium concentration, polyuria, thirst, and dehydration/hypovolaemia.

The oral LD50 of tolvaptan in rats and dogs is > 2000 mg/kg. No mortality was observed in rats or dogs following single oral doses of 2000 mg/kg (maximum feasible dose). A single oral dose of 2000 mg/kg was lethal in mice, and symptoms of toxicity in affected mice included decreased locomotor activity, staggering gait, tremor and hypothermia.

If overdose occurs, estimation of the severity of poisoning is an important first step. A thorough history and details of overdose should be obtained, and a physical examination should be performed. The possibility of multiple drug involvement should be considered.

Treatment should involve symptomatic and supportive care, with respiratory, ECG and blood pressure monitoring and water/electrolyte supplements as needed. A profuse and prolonged aquaresis should be anticipated, which, if not matched by oral fluid ingestion, should be replaced with intravenous hypotonic fluids, while closely monitoring electrolytes and fluid balance.

ECG monitoring should begin immediately and continue until ECG parameters are within normal ranges. Dialysis may not be effective in removing tolvaptan because of its high binding affinity for human plasma protein (> 99%). Close medical supervision and monitoring should continue until the patient recovers.

5 PHARMACOLOGICAL PROPERTIES

5.1 PHARMACODYNAMIC PROPERTIES

Tolvaptan is a Vasopressin antagonist: ATC code C03XA01.

Mechanism of action

Tolvaptan is a selective vasopressin V2-receptor antagonist with an affinity for the V2-receptor greater than that of native arginine vasopressin. When taken orally, 15 to 60 mg doses of SAMSCA tablets cause an increase in urine excretion resulting in increased aquaresis, decreased urine osmolality and increased serum sodium concentrations. Urine excretion of sodium and potassium are not significantly affected.

Tolvaptan metabolites do not appear to have relevant pharmacological activity at clinical concentrations in humans.

Oral administration of 15 to 120 mg doses of SAMSCA produced a significant increase in urine excretion rate within 2 hours of dosing. The increase in 24-hour urine volume was dose dependent. Following single oral doses of 15 to 60 mg, urine excretion rates returned to baseline levels after 24 hours. A mean of about 7 litres was excreted during 0 to 12 hours, independent of dose. Markedly higher doses of tolvaptan produce more sustained responses without affecting the magnitude of excretion, as active concentrations of tolvaptan are present for longer periods of time.

Clinical trials

In two double-blind, placebo-controlled, multi-center studies (SALT-1 and SALT-2), a total of 424 patients with euvolaemic or hypervolaemic hyponatraemia (serum sodium < 135 mmol/L) resulting from a variety of underlying causes (heart failure, liver cirrhosis, syndrome of inappropriate antidiuretic hormone [SIADH] and others) were treated for 30 days with tolvaptan or placebo, and were followed for an additional 7 days after withdrawal. Symptomatic patients, patients likely to require saline therapy during the course of therapy, patients with acute and transient hyponatremia associated with head trauma or postoperative state and patients with hyponatremia due to primary polydipsia, uncontrolled adrenal insufficiency or uncontrolled hypothyroidism were excluded. Patients were randomized to

receive either placebo (N = 220) or tolvaptan (N = 223) at an initial oral dose of 15 mg once daily.

The mean serum sodium concentration at study entry was 129 mmol/L. Fluid restriction was to be avoided if possible during the first 24 hours of therapy to avoid overly rapid correction of serum sodium. During the first 24 hours of therapy 87% of patients had no fluid restriction. Thereafter, patients could resume or initiate fluid restriction (defined as fluid intake of ≤ 1.0 liter/day) as clinically indicated.

The dose of tolvaptan could be increased at 24 hour intervals to 30 mg once daily, then to 60 mg once daily, until either the maximum dose of 60 mg or normonatreamia (serum sodium > 135 mmol/L) was reached. Serum sodium concentrations were determined at 8 hours after study drug initiation and daily up to 72 hours, within which time titration was typically completed. Treatment was maintained for 30 days with additional serum sodium assessments on Days 11, 18, 25 and 30. On the day of study discontinuation, all patients resumed previous therapies for hyponatraemia and were reevaluated 7 days later.

The primary endpoint for the studies was the average daily AUC for change in serum sodium from baseline to Day 4 and baseline to Day 30 in patients with a serum sodium less than 135 mmol/L. Compared to placebo, tolvaptan caused a statistically significant greater increase in serum sodium ($p < 0.0001$) during both periods in both studies (see Table 2). In addition, for patients with a serum sodium of < 130 mmol/L or < 125 mmol/L, the effects at Day 4 and Day 30 was also significant (see Table 2 below). This effect was also seen across all disease etiology subsets (e.g., CHF, cirrhosis, SIADH/other).

Table 2- Effects of Treatment with tolvaptan tablets 15 mg/day to 60 mg/day – pooled data from SALT-1 and SALT-2

	**Tolvaptan 15 mg/day to 60 mg/day**	**Placebo**	**Estimated Effect (95% CI)**
**Subjects with Serum Sodium < 135 mmol/L (ITT population)**			
Change in average daily serum [Na+] AUC baseline to Day 4 (mmol/L) Mean (SD) N[#]	4.0 (2.8) 213	0.4 (2.4) 203	3.7 (3.3-4.2) $p < 0.0001$
Change in average daily serum [Na+] AUC baseline to Day 30 (mmol/L) Mean (SD) N[#]	6.2 (4.0) 213	1.8 (3.7) 203	4.6 (3.9-5.2) $p < 0.0001$
Percent of Patients Needing Fluid Restriction*	14% 30/215	25% 51/206	$p < 0.01$
**Subgroup with Serum Sodium < 130 mmol/L**			
Change in average daily serum [Na+] AUC baseline to Day 4 (mmol/L) Mean (SD) N	4.8 (3.0) 110	0.7 (2.5) 105	4.2 (3.5-5.0) $p < 0.0001$

	**Tolvaptan 15 mg/day to 60 mg/day**	**Placebo**	**Estimated Effect (95% CI)**
Change in average daily serum [Na+] AUC baseline to Day 30 (mmol/L)<br>Mean (SD)<br>N	7.9 (4.1)<br>110	2.6 (4.2)<br>105	5.5 (4.4-6.5)<br>$p < 0.0001$
Percent of Patients Needing Fluid Restriction*	19%<br>21/110	36%<br>38/106	$p < 0.01$
**Subgroup with Serum Sodium < 125 mmol/L**			
Change in average daily serum [Na+] AUC baseline to Day 4 (mmol/L)<br>Mean (SD)<br>N	5.7 (3.8)<br>26	1.0 (1.8)<br>30	5.3 (3.8-6.9)<br>$p < 0.0001$
Change in average daily serum [Na+] AUC baseline to Day 30 (mmol/L)<br>Mean (SD)<br>N	10.0 (4.8)<br>26	4.1 (4.5)<br>30	5.7 (3.1-8.3)<br>$p < 0.0001$
Percent of Patients Needing Fluid Restriction*	35%<br>9/26	50%<br>15/30	$p = 0.14$

* Fluid Restriction defined as < 1 L/day at any time during treatment period.
# ITT Dataset

In patients with hyponatraemia (defined as < 135 mmol/L), serum sodium concentration increased to a significantly greater degree in tolvaptan -treated patients compared to placebo-treated patients as early as 8 hours after the first dose, and the change was maintained for 30 days. The percentage of patients requiring fluid restriction (defined as ≤ 1 L/day at any time during the treatment period) was also significantly less (p < 0.01) in the tolvaptan -treated group (30/215, 14%) as compared with the placebo-treated group (51/206, 25%).

Figure 1 shows the change from baseline in serum sodium by visit in patients with serum sodium < 135 mmol/L. Within 7 days of tolvaptan discontinuation, serum sodium concentrations in tolvaptan -treated patients declined to levels similar to those of placebo-treated patients.

Figure 1: Pooled SALT Studies: Analysis of Mean Serum Sodium (± SE, mmol/L) by Visit - Patients with Baseline Serum Sodium < 135 mmol/L

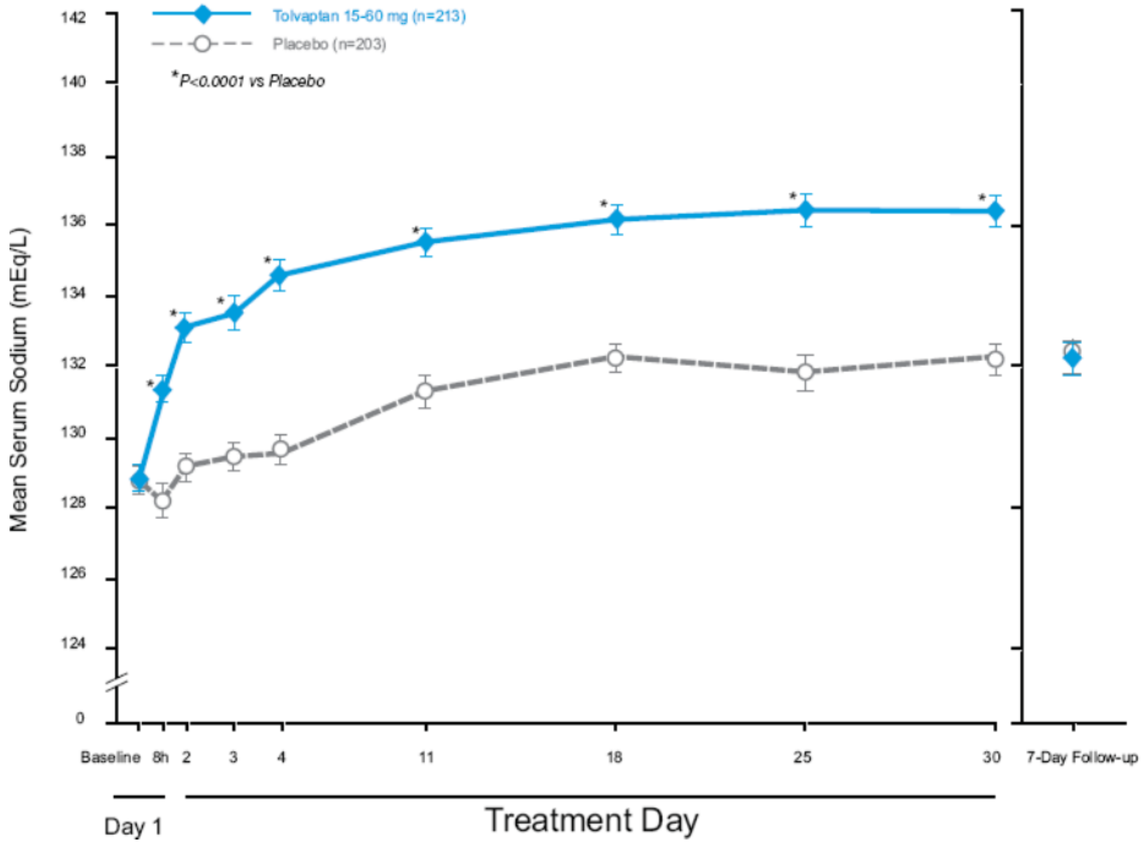

*p-value < 0.0001 for all visits during tolvaptan treatment compared to placebo

Figure 2: Pooled SALT Studies: Analysis of Mean Serum Sodium (± SE, mmol/L) by Visit - Patients with Baseline Serum Sodium < 130 mmol/L

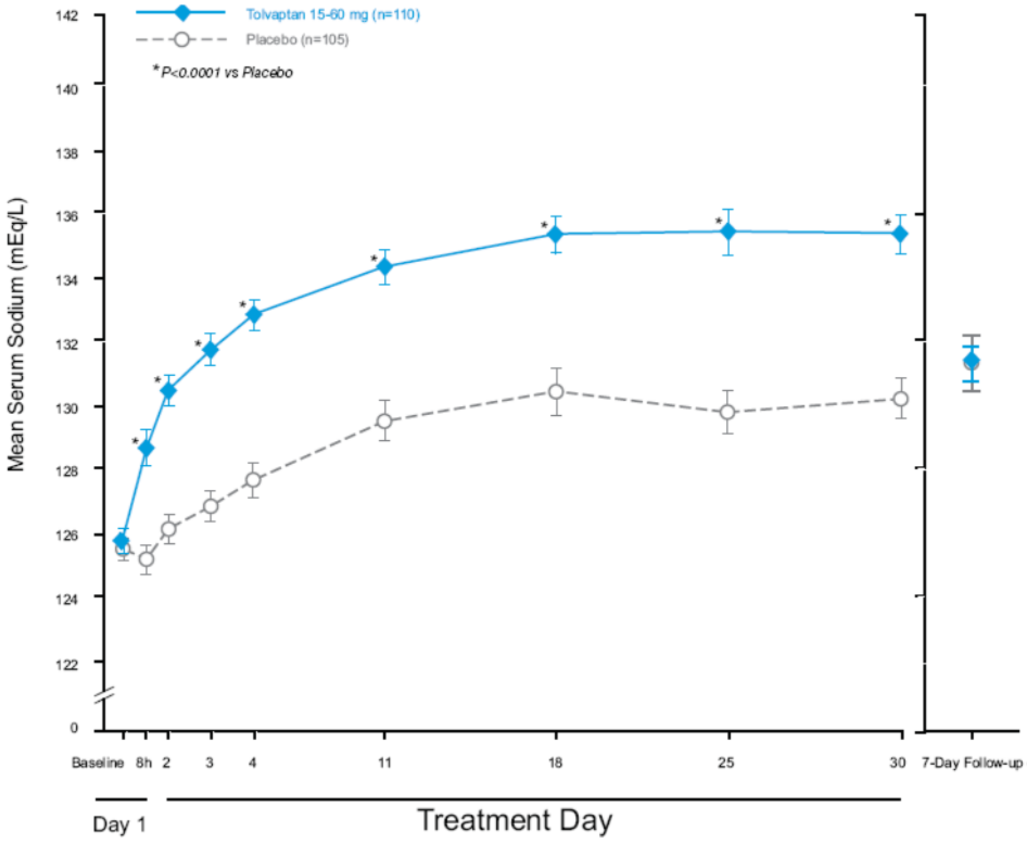

*p-value < 0.0001 for all visits during tolvaptan treatment compared to placebo

In the open-label study SALTWATER, 111 patients, 94 of them hyponatraemic (serum sodium < 135 mmol/L), previously on tolvaptan or placebo therapy were given SAMSCA as a titrated regimen (15 to 60 mg once daily) after having returned to standard care for at least 7 days. By this time, their baseline mean serum sodium concentration had fallen to between their original baseline and post-placebo therapy level. Upon initiation of therapy, average serum sodium concentrations increased to approximately the same levels as observed for those previously treated with tolvaptan, and were sustained for at least a year. Figure 3 shows results from 111 patients enrolled in the SALTWATER Study.

Figure 3: SALTWATER: Analysis of Mean Serum Sodium (± SE, mmol/L) by Visit

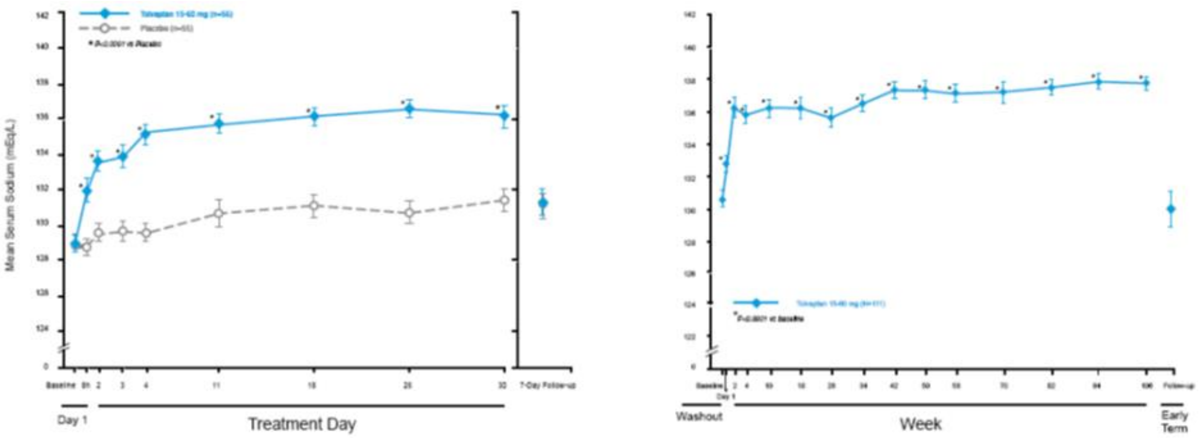

*p-value < 0.0001 for all visits during tolvaptan treatment compared to baseline

5.2 PHARMACOKINETIC PROPERTIES

Absorption and distribution

After oral administration, tolvaptan is rapidly absorbed with peak plasma concentrations occurring about 2 hours after dosing. The absolute bioavailability of tolvaptan is about 56%. Co-administration with food has no effect on plasma concentrations. Following single oral doses of ≥ 300 mg, peak plasma concentrations appear to plateau, possibly due to saturation of absorption. Tolvaptan binds reversibly (98%) to plasma proteins and is distributed into an apparent volume of distribution of about 3 L/kg.

Metabolism

Tolvaptan is extensively metabolised by the liver. Less than 1% of intact active substance is excreted unchanged in the urine. Radio-labelled tolvaptan experiments showed that 40% of the radioactivity was recovered in the urine and 59% was recovered in the faeces where unchanged tolvaptan accounted for 32% of radioactivity. Tolvaptan is only a minor component in plasma (3%). The terminal elimination half-life is about 8 hours and steady-state concentrations of tolvaptan are obtained after the first dose. After oral dosing, clearance is about 4 mL/min/kg.

Linearity

Tolvaptan has linear pharmacokinetics for doses of 15 to 60 mg.

5.3 PRECLINICAL SAFETY DATA

Genotoxicity

Tolvaptan tested negative for genotoxicity in *in vitro* (bacterial reverse mutation assay, mammalian forward mutation assay and chromosomal aberration test in Chinese hamster lung fibroblast cells) and *in vivo* (rat micronucleus assay) test systems.

Carcinogenicity

Up to two years of oral administration of tolvaptan to male and female rats at doses up to 1000 mg/kg/day (yielding 1.3- and 3.4-times respectively, the AUC for tolvaptan in patients at the maximum recommended human dose [MRHD]), to male mice at doses up to 60 mg/kg/day (relative exposure, 0.3) and to female mice at doses up to 100 mg/kg/day (relative exposure, 0.4) did not increase the incidence of tumours. The predictive value of these studies is limited by the inability to obtain high multiples of the clinical exposure to tolvaptan. However, negative findings in genotoxicity assays and the absence of pre-neoplastic lesions observed in these and other studies lend support to tolvaptan being unlikely to pose a particular carcinogenic risk in patients.

6 PHARMACEUTICAL PARTICULARS

6.1 LIST OF EXCIPIENTS

Maize starch
Hyprolose
Lactose monohydrate
Magnesium stearate
Microcrystalline cellulose
Indigo carmine aluminium lake

6.2 INCOMPATIBILITIES

Refer to Section 4.5 INTERACTIONS WITH OTHER MEDICINES AND OTHER FORMS OF INTERACTIONS.

6.3 SHELF LIFE

In Australia, information on the shelf life can be found on the public summary of the Australian Register of Therapeutic Goods (ARTG). The expiry date can be found on the packaging.

6.4 SPECIAL PRECAUTIONS FOR STORAGE

Store below 25°C.

Protect from light and moisture.

6.5 NATURE AND CONTENTS OF CONTAINER

SAMSCA is supplied as 15 mg and 30 mg tolvaptan tablets in PVC/aluminium perforated unit dose blister packs of 10 or 30 tablets each.

Not all presentations and pack sizes may be available in Australia.

6.6 SPECIAL PRECAUTIONS FOR DISPOSAL

In Australia, any unused medicine or waste material should be disposed of by taking to your local pharmacy.

6.7 PHYSICOCHEMICAL PROPERTIES

Tolvaptan is (±)-4'-[(7-chloro-2,3,4,5-tetrahydro-5-hydroxy-1*H*-1-benzazepin-1-yl) carbonyl]-*o*-tolu-*m*-toluidide. The empirical formula is $C_{26}H_{25}ClN_2O_3$. Molecular weight is 448.94.

Chemical structure

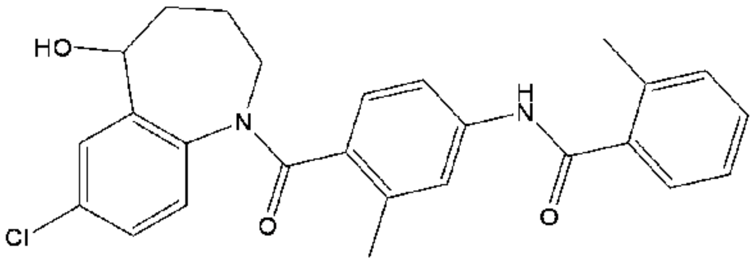

Tolvaptan is practically insoluble in water and the aqueous solubility of the drug substance is poor (~ 0.1 mg/250 mL) across all pH ranges. It is slightly soluble in ethyl acetate, sparingly soluble in ethanol, soluble in methanol and freely soluble in benzyl alcohol. The octanol:water partition coefficient was reported to be greater than 5000 at 25°C.

CAS number

The CAS Registry Number for tolvaptan is 150683-30-0.

7 MEDICINE SCHEDULE (POISONS STANDARD)

Schedule 4: Prescription Only Medicine (S4)

8 SPONSOR

Otsuka Australia Pharmaceutical Pty Ltd
Suite 2.03, Level 2
9 Help Street
Chatswood NSW 2067
www.otsuka.com.au

9 DATE OF FIRST APPROVAL

5th April 2012

10 DATE OF REVISION

26th March 2021

SUMMARY TABLE OF CHANGES

Section changed	Summary of new information
2	Addition of lactose statement
2, 6.7	Editorial changes

SAMSCA® is a registered trademark of Otsuka Pharmaceutical Co., Ltd.
