# Supplementary material for: Tolvaptan versus fluid restriction in acutely hospitalised patients with moderate-profound hyponatraemia (TVFR-HypoNa): design and implementation of an open-label randomised trial
Source: Trials. 2022 Apr 21;23:335. doi: 10.1186/s13063-022-06237-5 (PMC9028077; doi:10.1186/s13063-022-06237-5)
Supplement: Supplementary file 4 — Additional file 4: Appendix 4 [file 13063_2022_6237_MOESM4_ESM.pdf]

FAH018170

Place Patient Label Here

Participant Information Sheet/Consent Form

Interventional Study - *Adult providing own consent*

Austin Health

**Title**	Open-label randomised controlled trial of tolvaptan versus fluid restriction in acutely hospitalised patients with euvolaemic or hypervolaemic hyponatraemia
**Short Title**	Tolvaptan versus fluid restriction
**Protocol Number**	16
**Project Sponsor**	University of Melbourne
**Principal Investigators**	Prof Mathis Grossmann, Dr Nicholas Russell, Dr Annabelle Warren
**Location**	Austin Health

Part 1 What does my participation involve?

1 Introduction

You are invited to take part in this research project. This is because you have hyponatraemia. The research project is testing a new treatment for hyponatraemia. The new treatment is called tolvaptan.

This Participant Information Sheet/Consent Form tells you about the research project. It explains the tests and treatments involved. Knowing what is involved will help you decide if you want to take part in the research.

Please read this information carefully. Ask questions about anything that you don't understand or want to know more about. Before deciding whether or not to take part, you might want to talk about it with a relative, friend or your local doctor.

Participation in this research is voluntary. If you don't wish to take part, you don't have to. You will receive the best possible care whether or not you take part.

If you decide you want to take part in the research project, you will be asked to sign the consent section. By signing it you are telling us that you:
- Understand what you have read
- Consent to take part in the research project
- Consent to have the tests and treatments that are described
- Consent to the use of your personal and health information as described.

You will be given a copy of this Participant Information and Consent Form to keep.

2 What is the purpose of this research?

Hyponatraemia (low blood sodium salt concentration) is common in hospitalised patients. It can cause symptoms such as nausea, unsteadiness, headache, and confusion.

The main treatment for hyponatraemia is restricting the amount of fluid the patient drinks, in order to raise the blood sodium concentration. This approach is sometimes not very effective, and it can take a long time to work. Fluid restriction can also cause thirst and discomfort for patients. Tolvaptan is a relatively new drug that we think might be better at treating hyponatraemia than fluid restriction.

Tolvaptan has been proven to work outside the hospital in patients with mild hyponatraemia. We think it might also be more effective than fluid restriction for hospital inpatients and this trial is designed to test whether that is the case.

Medications, drugs and devices have to be approved for use by the Australian Federal Government. Tolvaptan is approved in Australia, including for the type of use in this study.

This research has been initiated by the study doctors, Professor Mathis Grossmann and Dr Nicholas Russell.

This research has been partly-funded by a grant from Otsuka Pharmaceuticals.

3 What does participation in this research involve?

3.1 Study Design

You will be participating in a randomised controlled research project. Sometimes we do not know which treatment is best for treating a condition. To find out we need to compare different treatments. We put people into groups and give each group a different treatment. The results are compared to see if one is better. To try to make sure the groups are the same, each participant is put into a group by chance (random). You will have a one in two chance of receiving tolvaptan and a one in two chance of receiving usual care (fluid restriction).

This research project has been designed to make sure the researchers interpret the results in a fair and appropriate way and avoids study doctors or participants jumping to conclusions.

3.2 Details of your involvement

We would need your agreement and signature on the consent form before doing any study assessments.

After this, the initial steps will take about 20 minutes. These are:

- Interview. One of our doctors will ask about your medical history and the medications you are taking. We can get most of this information from your Austin Health medical record.
- Blood test. These are normal blood tests involving a small needle in your arm. We are checking electrolytes (blood salts), glucose, liver function, kidney function, and hormone levels (cortisol and thyroid stimulating hormone). Even if you weren't in the trial, you would be having blood tests at this stage in your care.
- Urine sample. This will be taken once per day in the morning. Even if you were not in the trial, these urine samples would be taken to monitor your urine sodium concentration.

- Measurements. We will be noting your blood pressure, weight, and heart rate. Even if you weren't in the trial, these would be measured routinely.
- Timed Up and Go Test. This is a test of mobility. It involves seeing how long it takes you to get up from a chair, walk 3 metres, return to the chair, and sit down.

We will review the results of these assessments to make sure that there isn't a problem with you participating in the trial. Certain medical conditions mean that we would not be able to include you. If you are not eligible to be in the trial, you will continue with usual care from your treating medical team.

If you are eligible, then you will be randomly assigned to tolvaptan treatment or usual care (fluid restriction). You will know which treatment you are assigned to. If you are assigned to tolvaptan treatment, this is given as a once a day oral dose for 3 days. The first dose is either 7.5mg or 15mg, depending on whether you have detectable extra fluid in your body. On day 2 and day 3, the dose depends on what has happened to your blood sodium level in the previous 24 hours. The dose will be either 0mg (no dose), 15mg, 30mg, or 60mg. Tolvaptan will be dispensed by Austin Health Clinical Trials Pharmacy and administered to you by Austin Health nursing staff. If you are assigned to usual care, your oral fluid intake will be limited to 1500mL for the first day. On day 2 and day 3, the fluid restriction depends on what has happened to your blood sodium level in the previous 24 hours. The fluid restriction will range from no fluid restriction to complete fasting (no fluid allowed at all). If your blood sodium level rises too quickly, you will be given an intravenous drip of water to prevent any further rise. This would occur even if you were not in the trial.

During your participation in the 4-day trial the following assessments will be made:

- Blood test. This will occur every 4 hours until midnight on day 1, and then every 6 hours. Even if you were not in the trial, these blood samples would be taken to monitor your blood sodium concentration. This will take about 5 minutes each time.
- Urine sample. This will be taken once per day in the morning. Even if you were not in the trial, these urine samples would be taken to monitor your urine sodium concentration.
- Measurements. Your blood pressure, heart rate, and weight will be recorded each morning. Even if you were not in the trial, these measurements would be made as part of routine nursing care. This will take about 5 minutes each time.
- Timed Up and Go Test. This will be done once per day. This is a test of mobility. It involves seeing how long it takes you to get up from a chair, walk 3 metres, return to the chair, and sit down. This will take about 1 minute each time.
- Questionnaires. One of the study doctors will ask you about symptoms of hyponatraemia. This will take about 15 minutes each time and will occur once daily.

When you leave hospital, one of the study doctors will fill out a final questionnaire with you. This will take about 10 minutes.

On the 34th day after you commence the study, one of the study doctors will ring you to fill out a final questionnaire with you. This will take about 10 minutes. You will also be asked to have a blood test to check your blood sodium concentration one final time. Even if you were not in the trial, this blood test would be taken to monitor your blood sodium concentration.

There are no additional costs associated with participating in this research project, nor will you be paid. All medication, tests and medical care required as part of the research project will be provided to you free of charge.

4 What do I have to do?

To participate in this study you will need to undergo the study assessments listed above. If you are assigned to tolvaptan treatment, you will take one dose of tolvaptan per day for 3 days. You will not be fluid restricted. If you are assigned to fluid restriction the amount you are permitted to drink will be restricted for 3 days and you will not be given tolvaptan. After 3 days, the study intervention ceases, and treatment for your hyponatraemia if it still present will be up to your treating medical team.

If you are ready to go home before 3 days, the study will cease at the time you are discharged. You will not be asked to return for any study assessments after hospital discharge. A study doctor will ring you to complete a final questionnaire on day 34.

5 Other relevant information about the research project

This study is only being conducted at Austin Health. We aim to enrol a total of 166 participants (83 in the tolvaptan group and 83 in the fluid restriction group).

6 Do I have to take part in this research project?

Participation in any research project is voluntary. If you do not wish to take part, you do not have to. If you decide to take part and later change your mind, you are free to withdraw from the project at any stage.

If you do decide to take part, you will be given this Participant Information and Consent Form to sign and you will be given a copy to keep.

Your decision whether to take part or not to take part, or to take part and then withdraw, will not affect your routine treatment, your relationship with those treating you or your relationship with Austin Health.

7 What are the alternatives to participation?

You do not have to take part in this research project to receive treatment at this hospital. Other options are available; these include usual care, which generally involves fluid restriction. Your study doctor will discuss this option with you before you decide whether or not to take part in this research project. You can also discuss the options with your treating medical team.

8 What are the possible benefits of taking part?

We cannot guarantee or promise that you will receive any benefits from this research; however, possible benefits may include faster resolution of your hyponatraemia. We hope that we can prove that tolvaptan is better than fluid restriction in which case it will be used more often in the treatment of hyponatraemia for future patients.

9 What are the possible risks and disadvantages of taking part?

Medical treatments often cause side effects. You may have none, some or all of the effects listed below, and they may be mild, moderate or severe. If you have any of these side effects, or

are worried about them, talk with your study doctor. Your study doctor will also be looking out for side effects.

There may be side effects that the researchers do not expect or do not know about and that may be serious. Tell your study doctor immediately about any new or unusual symptoms that you get.

Many side effects go away shortly after treatment ends. However, sometimes side effects can be serious, long lasting or permanent. If a severe side effect or reaction occurs, your study doctor may need to stop your treatment. Your study doctor will discuss the best way of managing any side effects with you.

Side Effect	How often is it likely to occur?	How severe might it be?	How long might it last?
Thirst or dry mouth	1 in 10	Minor	For the duration of treatment only
Feeling of weakness	1 in 10	Minor	For the duration of treatment only
Constipation	1 in 10	Minor	For the duration of treatment only
Passing more urine	1 in 10	Minor	For the duration of treatment only
High blood sugar	1 in 20	Minor	For the duration of treatment only

Any treatment for hyponatraemia needs to strike a balance. On one hand slow correction of hyponatraemia is harmful because it can prolong symptoms, length of hospital stay, and, if the hyponatraemia is severe, fail to prevent death from hyponatraemia. On the other hand, correction that is much too fast can cause a serious permanent brain injury. This is extremely rare. There have been no reports of this happening when tolvaptan has been used as described in this trial.

Having a blood sample taken may cause some discomfort, bruising, minor infection or bleeding. If this happens, it can be easily treated.

Any side effects from your involvement in this trial would be managed as part of your normal care at Austin Health. If you elect to be treated as a public patient this would be free of charge.

10 What will happen to my test samples?

A mandatory component of this research is the collection of blood and urine samples. This is for the purpose of measuring blood and urine sodium concentration, and other parameters relevant to hyponatraemia such as kidney and liver function, thyroid function, and cortisol concentrations.

Blood and urine samples in this study are handled and processed in the same way as routine samples collected as part of usual hospital care. These samples are individually identifiable because they are labelled with your name, date of birth, address, and hospital number. After collection they are sent to Austin Pathology on site and processed in the usual way. The results are uploaded into your file on the hospital electronic record system. The samples are then destroyed by Austin Pathology after 7 days.

11 What if new information arises during this research project?

Sometimes during the course of a research project, new information becomes available about the treatment that is being studied. If this happens, your study doctor will tell you about it and discuss with you whether you want to continue in the research project. If you decide to withdraw, your study doctor will make arrangements for your regular health care to continue. If you decide to continue in the research project you will be asked to sign an updated consent form.

Also, on receiving new information, your study doctor might consider it to be in your best interests to withdraw you from the research project. If this happens, he/ she will explain the reasons and arrange for your regular health care to continue.

12 Can I have other treatments during this research project?

Whilst you are participating in this research project, you may not be able to take some or all of the medications or treatments you have been taking for your condition or for other reasons. It is important to tell your study doctor and the study staff about any treatments or medications you may be taking, including over-the-counter medications, vitamins or herbal remedies, acupuncture or other alternative treatments. You should also tell your study doctor about any changes to these during your participation in the research project. Your study doctor should also explain to you which treatments or medications need to be stopped for the time you are involved in the research project.

13 What if I withdraw from this research project?

If you decide to withdraw from the project, please notify a member of the research team before you withdraw. This notice will allow that person or the research supervisor to discuss any health risks or special requirements linked to withdrawing.

If you do withdraw your consent during the research project, the study doctor and relevant study staff will not collect additional personal information from you, although personal information already collected will be retained to ensure that the results of the research project can be measured properly and to comply with law. You should be aware that data collected by the sponsor up to the time you withdraw will form part of the research project results. If you do not want them to do this, you must tell them before you join the research project.

14 Could this research project be stopped unexpectedly?

This research project may be stopped unexpectedly for a variety of reasons. These may include reasons such as:

- Unacceptable side effects
- The drug being shown not to be effective
- The drug being shown to work and not need further testing
- Decisions made by local regulatory/health authorities.

15 What happens when the research project ends?

When this research project ends, your care will continue as judged most appropriate by your treating medical team. Tolvaptan is not in routine use for hospital patients with hyponatraemia, so at the end of the study, if you were to remain hyponatraemic, it is likely your treating medical team would stop this drug and switch you to a more usual type of treatment (most often this is fluid restriction).

On completion of the research project, there will be a period of analysis of results. You will then be sent a letter explaining the results of the research project.

Part 2 How is the research project being conducted?

16 What will happen to information about me?

By signing the consent form you consent to the study doctor and relevant research staff collecting and using personal information about you for the research project. Any information obtained in connection with this research project that can identify you will remain confidential. Paper copy information will be stored in a locked filing cabinet in a locked area of the Department of General Medicine. For the purposes of stored information, participants will be identified by study number, name, date of birth, and address. This is to allow 3-point identification of study participants. The only other identifying information stored in these files will be contact phone number, to allow the Day 34 exit interview to take place by phone. An electronic database file will also be kept containing the same study information, as a back-up and as a way to analyse this information. This will be stored on a password-protected file on an Austin Health server. Only study personnel will have access to this information. Your information will only be used for the purpose of this research project and it will only be disclosed with your permission, except as required by law. Results of your blood and urine tests will be uploaded into your electronic health record at Austin Health. These results are accessible to any clinician involved in your management at Austin Health.

Information about you may be obtained from your health records held at this and other health services for the purpose of this research. By signing the consent form you agree to the study team accessing health records if they are relevant to your participation in this research project.

Your health records and any information obtained during the research project are subject to inspection (for the purpose of verifying the procedures and the data) by the relevant authorities and authorised representatives of the Sponsor, University of Melbourne, the institution relevant to this Participant Information Sheet, Austin Health, or as required by law. By signing the Consent Form, you authorise release of, or access to, this confidential information to the relevant study personnel and regulatory authorities as noted above.

It is anticipated that the results of this research project will be published and/or presented in a variety of forums. In any publication and/or presentation, information will be provided in such a way that you cannot be identified, except with your permission. This can be guaranteed because in any publication or presentation, results will be published in aggregate only, and contain no way of identifying individual participants. Some medical journals require the additional publication of individual study participant results. In any such publication, your individual results would be identified by a code only and contain no information which would allow people outside of this research project to link them to you.

Information about your participation in this research project may be recorded in your health records.

In accordance with relevant Australian and/or Victorian privacy and other relevant laws, you have the right to request access to your information collected and stored by the research team. You also have the right to request that any information with which you disagree be corrected. Please contact the study team member named at the end of this document if you would like to access your information.

Any information obtained for the purpose of this research project that can identify you will be treated as confidential and securely stored. It will be disclosed only with your permission, or as required by law. Electronic and paper study files will be kept for 15 years at which point they will be securely destroyed.

17 Complaints and compensation

17.1 Complaints

If you have a complaint about any aspect of your medical care or treatment at Austin Health please raise this with your doctor directly. If this is not possible or does not resolve the complaint then the Centre for Patient Experience will help you. You can speak to a consumer liaison officer at the Centre for Patient Experience by calling (03) 9496 3566.

17.2 Treatment Available

If you suffer any injuries or complications as a result of this research project, you should contact the study team as soon as possible and you will be assisted with arranging appropriate medical treatment. If you are eligible for Medicare, you can receive any medical treatment required to treat the injury or complication, free of charge, as a public patient in any Australian public hospital.

17.3 Compensation

In the unlikely event that you suffer an injury as a result of participating in this trial, hospital care and treatment will be provided by the public health service at no extra cost to you.

Neither the hospital nor the investigators guarantee that compensation for other loss or injury will be available to you (such as loss of income from work days missed or health care costs not covered by public health services). However, by signing the consent form, you have not waived any legal or other right to seek compensation, including legal rights for negligence or other causes of action.

18 Who is organising and funding the research?

This research project is being conducted by Professor Mathis Grossmann and Dr Nicholas Russell. It is being supported by funding to the University of Melbourne from Otsuka Australia Pharmaceuticals.

Otsuka Australia Pharmaceuticals may benefit financially from this research project if, for example, the project produces results which helps Otsuka Australia Pharmaceuticals to sell more tolvaptan.

You will not benefit financially from your involvement in this research project even if, for example, knowledge acquired from analysis of your samples proves to be of commercial value to Otsuka Australia Pharmaceuticals.

In addition, if knowledge acquired through this research leads to discoveries that are of commercial value to Otsuka Australia Pharmaceuticals, the study doctors or their institutions, there will be no financial benefit to you or your family from these discoveries.

University of Melbourne will receive a payment from Otsuka Australia Pharmaceuticals for undertaking this research project. No member of the research team will receive a personal financial benefit from your involvement in this research project (other than their ordinary wages).

19 Who has reviewed the research project?

All research in Australia involving humans is reviewed by an independent group of people called a Human Research Ethics Committee (HREC). The ethical aspects of this research project have been approved by the HREC of Austin Health.

This project will be carried out according to the *National Statement on Ethical Conduct in Human Research (2007)*. This statement has been developed to protect the interests of people who agree to participate in human research studies.

20 Further information and who to contact

The person you may need to contact will depend on the nature of your query.

If you want any further information concerning this project or if you have any medical problems which may be related to your involvement in the project (for example, any side effects), you can contact the principal study doctors on (03) 9496 5000 or at any time call:

Clinical contact person

Position	On Call Endocrinology Registrar
Telephone	(03) 9496 5000

If you have any complaints about any aspect of the project, the way it is being conducted or any questions about being a research participant in general, then you may contact:

Complaints contact person

Position	Complaints Officer
Telephone	(03) 9496 4090 or (03) 9496 4035
Email	ethics@austin.org.au

If you need to contact the Human Research Ethics Committee that approved this project, then you may contact:

Reviewing HREC and HREC Executive Officer

Reviewing HREC name	Austin Health Human Research Ethics Committee
HREC Executive Officer	Mrs Lisa Pedro
Telephone	(03) 9496 4035
Email	ethics@austin.org.au

Consent Form - *Adult providing own consent*

**Title**	Open-label randomised controlled trial of tolvaptan versus fluid restriction in acutely hospitalised patients with euvolaemic or hypervolaemic hyponatraemia
**Short Title**	Tolvaptan versus fluid restriction
**Protocol Number**	15
**Project Sponsor**	University of Melbourne
**Principal Investigators**	Prof Mathis Grossmann, Dr Nicholas Russell
**Location**	Austin Health

Consent Agreement

I have read the Participant Information Sheet or someone has read it to me in a language that I understand.

I understand the purposes, procedures and risks of the research described in the project.

I give permission for my doctors, other health professionals, hospitals or laboratories outside this hospital to release information to Austin Health concerning my disease and treatment for the purposes of this project. I understand that such information will remain confidential.

I have had an opportunity to ask questions and I am satisfied with the answers I have received.

I freely agree to participate in this research project as described and understand that I am free to withdraw at any time during the study without affecting my future health care.

I understand that I will be given a signed copy of this document to keep.

Declaration by Participant – for participants who have read the information

Name of Participant (please print) ____

Signature ________________________________ Date ________________________________

Declaration - for participants unable to read the information and consent form

Witness to the informed consent process
Name (please print) ____

Signature ________________________________ Date ________________________________
* Witness must be 18 years or older.

Declaration by Study Doctor/Senior Researcher†

I have given a verbal explanation of the research project, its procedures and risks and I believe that the participant has understood that explanation.

Name of Study Doctor/
Senior Researcher† (please print) ____

Signature ________________________________ Date ________________________________

† A senior member of the research team must provide the explanation of, and information concerning, the research project.
Note: All parties signing the consent section must date their own signature.

Form for Withdrawal of Participation - *Adult providing own consent*

**Title**	Open-label randomised controlled trial of tolvaptan versus fluid restriction in acutely hospitalised patients with euvolaemic or hypervolaemic hyponatraemia
**Short Title**	Tolvaptan versus fluid restriction
**Protocol Number**	15
**Project Sponsor**	University of Melbourne
**Principal Investigators**	Prof Mathis Grossmann, Dr Nicholas Russell
**Location**	Austin Health

Declaration by Participant

I wish to withdraw from participation in the above research project and understand that such withdrawal will not affect my routine treatment, my relationship with those treating me or my relationship with Austin Health.

Name of Participant (please print) ______________________________

Signature ______________________ Date ______________________

In the event that the participant's decision to withdraw is communicated verbally, Study Doctor/Senior Researcher to provide a description of the circumstances below.

Declaration by Study Doctor/Senior Researcher†

I have given a verbal explanation of the implications of withdrawal from the research project and I believe that the participant has understood that explanation.

Name of Study Doctor/
Senior Researcher† (please print) ______________________________

Signature ______________________ Date ______________________

† A senior member of the research team must provide the explanation of and information concerning withdrawal from the research project.

Note: All parties signing the consent section must date their own signature.
