# Supplementary material for: Tolvaptan versus fluid restriction in acutely hospitalised patients with moderate-profound hyponatraemia (TVFR-HypoNa): design and implementation of an open-label randomised trial
Source: Trials. 2022 Apr 21;23:335. doi: 10.1186/s13063-022-06237-5 (PMC9028077; doi:10.1186/s13063-022-06237-5)
Supplement: Supplementary file 1 — Additional file 1: Appendix 1. Austin Health Human Research Ethics Committee Approval Letter [file 13063_2022_6237_MOESM1_ESM.pdf]

Austin Hospital

145 Studley Road
PO Box 5555 Heidelberg
Victoria Australia 3084
Telephone 03 9496 5000
Facsimile 03 9458 4779
www.austin.org.au

SITE SPECIFIC ASSESSMENT (SSA) AUTHORISATION

APPROVAL TO CONDUCT A NEW RESEARCH PROJECT AT AUSTIN HEALTH

Dr. Nicholas Russell,
Departments of General Medicine and Endocrinology,

28 May 2021

Dear Dr. Nicholas Russell,

HREC Reference Number: HREC/48055/Austin-2019

Austin Health SITE REFERENCE Number: DT 48055/2019

Project Title: Open-label randomised controlled trial of tolvaptan versus fluid restriction in acutely hospitalised patients with euvolaemic or hypervolaemic hyponatraemia.

SSA Reference Number: SSA/48055/Austin-2019

Reviewing HREC: Austin Health Human Research Ethics Committee (EC00204)

HREC Approval Date: 15/12/2020

SSA Authorisation Date: 28 May 2021

I am pleased to advise that the above project satisfy Austin Health's governance requirements and may now be conducted at Austin Health. Conduct of the project is subject to compliance with the conditions set out below and any additional conditions specified by the reviewing HREC.

SSA Approved Documents:

Document	Version	Date
Austin Health HREC approval letter, including all listed approved documentation	-	15 December 2020
Clinical Trial Research Agreement between Austin Health and University of Melbourne	-	26 May 2021

Research governance

Condition of Governance Approval:

P. 03 9496 5000
F. 03 9458 4779
austin.org.au
145 Studley Road
Heidelberg
Victoria 3084
PO Box 5555
Heidelberg
Victoria 3084

1. Researchers must comply with the Investigator's Responsibilities in Research Procedure and Good Clinical Practice (ICH GCP). The Principal Research is to ensure that all associate researchers are aware of terms of approval and to ensure the project is conducted as specified in the application and in accordance with the National Statement on Ethical Conduct in Human Research (2007, updated 2018).
2. The Principal Investigator must notify the 1) CPI, 2) Reviewing Human Research Ethics Committee (RHREC) and Sponsor (if applicable) of:
   - All related internal Serious Adverse Events (SAE) in accordance with the NHMRC Position Statement: Safety *Monitoring and reporting in clinical trials involving therapeutic goods November 2016*.
   - Any other serious adverse effects to or complaints from Austin Health participants and steps taken to deal with them
   - Your inability to continue as Principal Investigator
   - Any unexpected developments in the project with ethical implications
   - Notify the RHREC of the failure to commence the study within 12 months of the RHREC approval date or if a decision is taken to end the study at any of the sites prior to the expected date of completion.
3. You are required to inform the Research Governance Office of;
   - The actual start date of the project at Austin Health
   - Any other matters which may impact the conduct of the project at Austin Health
   - Austin Health Investigators withdrawing from or joining the project.
4. Any amendments submitted to and approved by the RHREC, including changes to the protocol, approved documents and/or the addition of documents to be used at Austin Health, must be submitted for governance approval prior to implementation. After RHEC approval, the PI must submit a copy of all documents relating to the approved amendment, along with the RHREC approval certificate, to the Research Governance Office for approval.
5. Any changes to the indemnity, insurance arrangements or Clinical Trial Research Agreement for this project. This includes changes to the project budget or other changes, which may have financial or other resource implications for Austin Health.
6. RHREC approval must remain current for the entire duration of the project. Investigators undertaking projects without current RHREC approval risk their indemnity, funding and publication rights.
7. **If your project involves radiation:**
   - **It is your responsibility to ensure the research is added to the site Management Licence issued by Department of Human Services – Radiation Safety Section prior to study commencement should it be required (check your Medical Physicist Report). The site RGO must be notified when the research has been added to the licence.**
   - **You are legally obliged to conduct your research in accordance with the Australian Radiation Protection and Nuclear Safety Agency Code of Practice 'Exposure of Humans to Ionizing Radiation for Research Purposes' Radiation Protection series Publication No.8 (May 2005)(ARPANSA Code).**

Clinical Trial projects:

8. For clinical trials where Austin Health is the Sponsor, you are required to contact the Research Governance Office to organise submission of the CTN to the TGA. <u>This must be completed before commencement of your project.</u>

P. 03 9496 5000
F. 03 9458 4779
austin.org.au
145 Studley Road
Heidelberg
Victoria 3084
PO Box 5555
Heidelberg
Victoria 3084

9. Prior to commencement of the project, a copy of the governance authorisation letter and CTN acknowledgement must be provided to the Clinical Trials Pharmacy.
10. It is the Principal Investigator's responsibility to ensure they receive a copy of the submitted clinical trial notification acknowledgement letter for their site.

You are also required to submit to the Office for Research:

11. In addition to the reporting requirements of the RHREC, you are required to submit an Annual Progress Report for the duration of the project. A copy of this report should also be submitted to the CPI. Continuation of SSA approval is contingent on submission of an annual report, due within one month of the approval anniversary. Continued SSA and HREC approval are contingent on receipt of an annual report by the RHREC and the Research Governance Office.
12. A comprehensive Final Report upon completion of the project.

The Office for Research may conduct an audit of the project at any time.

Yours sincerely,

Priyanka Sathe

Digitally signed by Priyanka Sathe
Date: 2021.05.28 15:41:26 +10'00'

Priyanka Sathe
Acting Manager – Ethics & Research Governance
Office for Research, Austin Health, Level 8 HSB.
E-mail: ethics@austin.org.au
Web: http://www.austin.org.au/researchethics

P. 03 9496 5000
F. 03 9458 4779
austin.org.au

145 Studley Road
Heidelberg
Victoria 3084

PO Box 5555
Heidelberg
Victoria 3084
